# Scalability of spheroid-derived small extracellular vesicles production in stirred systems

**DOI:** 10.3389/fbioe.2025.1516482

**Published:** 2025-04-29

**Authors:** Thibaud Dauphin, Laurence de Beaurepaire, Apolline Salama, Quentin Pruvost, Clémentine Claire, Karine Haurogné, Sophie Sourice, Aurélien Dupont, Jean-Marie Bach, Julie Hervé, Eric Olmos, Steffi Bosch, Blandine Lieubeau, Mathilde Mosser

**Affiliations:** ^1^ Oniris VetAgroBio, INRAE, IECM, Nantes, France; ^2^ Oniris VetAgroBio, B-FHIT, Nantes, France; ^3^ CNRS, INSERM, BIOSIT_UAR 3480, Univ Rennes, Inserm 018, Rennes, France; ^4^ University of Lorraine, CNRS, LRGP, Nancy, France

**Keywords:** spheroid, extracellular vesicles, bioreactor, bioproduction, bioprocess, scale-up, shear stress

## Abstract

**Introduction:**

Small extracellular vesicle (sEV)-based therapies have gained widespread interest, but challenges persist to ensure standardization and high-scale production. Implementing upstream processes in a chemically defined media in stirred-tank bioreactors (STBr) is mandatory to closely control the cell environment, and to scale-up production, but it remains a significant challenge for anchorage-dependent cells.

**Methods:**

We used a human β cell line, grown as monolayer or in suspension as spheroid in stirred systems. We assessed the consequences of culturing these cells in 3D with, or without fetal bovine serum in a chemically defined medium, for cell growth, viability and metabolism. We next explored how different scale-up strategies might influence cell and spheroid formation in spinner flask, with the aim to transfer the process in instrumented Ambr®250 STBr. Lastly, we analyzed and characterized sEV production in monolayer, spinner flask and STBr.

**Results and discussion:**

Generation of spheroids in a chemically defined medium allowed the culture of highly viable cells in suspension in stirred systems. Spheroid size depended on the system’s volumetric power input (P/V), and maintaining this parameter constant during scale-up proved to be the optimal strategy for standardizing the process. However, transferring the spinner flask (SpF) process to the Ambr®250 STBr at constant P/V modified spheroid size, due to important geometric differences and impeller design. Compared to a monolayer reference process, sEV yield decreased two-fold in SpF, but increased two-fold in STBr. Additionally, a lower expression of the CD63 tetraspanin was observed in sEV produced in both stirred systems, suggesting a reduced release of exosomes compared to ectosomes. This study addresses the main issues encountered in spheroid culture scale-up in stirred systems, rather conducive for the production of ectosomes.

## 1 Introduction

Extracellular vesicles (EV) are membrane nanoparticles naturally released by all cell types. They comprise a variety of soluble and membrane-bound proteins, nucleic acids, and lipids, and play significant roles in intercellular communication. Three major subtypes of EV are described: exosomes (50–200 nm) originating from endosomal compartments and released via multivesicular bodies upon fusion to the plasma membrane, ectosomes (50–1,000 nm), and apoptotic bodies (100–5,000 nm), respectively released by budding and blebbing of the plasma membrane (van Niel et al., 2018). Conventionally, reference is made to small (<200 nm, sEV) or large (>200 nm) EV due to the limitations of current isolation techniques in separating each subtype (Welsh et al., 2024). As natural carriers of biological information, and non-replicative, the therapeutic potential of EV, particularly the sEV sub-populations, has garnered growing interest (Silva et al., 2021). Native or engineered EV are promising therapeutic tools in a broad range of regenerative medicines, autoimmune, infectious, neurodegenerative diseases, and cancers (Herrmann et al., 2021), with several clinical trials ongoing (Fusco et al., 2024).

Beyond the regulatory aspects, transferring EV as a novel category of biological medicines to the clinics, requires large-scale and standardized production. Studies in mice use 0.008–27 mg/kg of EV to observe therapeutic effects (Varderidou-Minasian and Lorenowicz, 2020), and based on an average yield of 2.5 µg of EV-associated protein per 10^6^ producing cells, calculated from 54 publications (Gudbergsson et al., 2016), treating a 70 kg human would require the production of 10^8^ to 10^11^ cells. To date, several strategies have been explored to enhance EV yields, including physical, chemical, or pharmacological preconditioning of the producing cells (Erwin et al., 2023). However, the cellular environment and the attributes of raw materials must be carefully controlled to ensure the targeted quality of EV and standardize the process, as these parameters can significantly affect EV phenotype and purity. In this context, culturing cells in chemically defined media in controlled bioreactors has emerged as the optimal strategy for the scalable production of EV while closely regulating the cell environment.

For anchorage-dependent cells, most studies have produced cells in standard culture flasks, which are not compatible with large-scale production. Several teams have successfully produced sEV in hollow-fiber bioreactors (HFB), which offer a surface area of up to 2.1 m^2^ for the Quantum^®^ HFB from Terumo. Cells could grow at high density and form a 3D network between fibers and could remain viable for months in a continuously perfused medium, while sEV accumulating in the cell compartment can be regularly harvested. sEV production in HFB have been described to improve yield and properties compared to a reference monolayer (ML) process in tissue culture flasks (T-flasks) (Watson et al., 2016; Cao et al., 2020; Yan and Wu, 2020). However, the HFB setup can create nutrient gradients and does not allow direct access to the cells for external monitoring. Yet, the main drawback of this system is its lack of scalability (Pincela Lins et al., 2023). STBr are considered more suitable for monitoring cell culture and allowing more precise control of mass and gas transfer, which is essential for proper scale-up. However, establishing culture in STBr remains a significant challenge for anchorage-dependent cells, which include most of the cells used for therapeutic EV production, such as mesenchymal stem cells (MSC).

Using microcarriers, which are polymer beads of 100–300 µm, is a common method to culture anchorage-dependent cells in suspension and has been successfully employed to produce sEV (Casajuana Ester and Day, 2023). However, since microcarriers rely on cell adhesion to exogenous materials the wide variety of raw materials, shape, size, and porosity makes selecting the appropriate microcarrier challenging. Moreover, there are concerns that microcarrier materials might be co-isolated with EV during downstream processing. An alternative approach relies on the inherent tendency of anchorage-dependent cells to self-agglomerate as spheroids in non-adhesive surface environments. The three-dimensional organization, enabled by intercellular contacts within spheroids, provides a more physiological environment than conventional 2D culture, resulting in enhanced cell functionality. Indeed, the morphology, organization, polarity, as well as phenotypic and functional characteristics of cells within spheroids are more representative of their native tissues (Laschke and Menger, 2017). In agreement, spheroid-derived sEV were shown to exhibit “*in vivo*-like” phenotypes (Thippabhotla et al., 2019; Tu et al., 2021). As far as stem cells are concerned, spheroid culture increases their differentiation capacity and enhances their regenerative and immunomodulatory properties (Cesarz and Tamama, 2016). The sEV released by MSC grown as spheroids also displayed higher regenerative capacity and antioxidant activity *in vitro* (Yuan et al., 2022). Interestingly, Zhang et al. also reported improved anti-inflammatory properties *in vivo* associated with a modification of the spheroid-derived sEV proteome and transcriptome, notably enriched in anti-inflammatory miRNAs (Zhang et al., 2021). Similarly, Hu et al. observed enrichment of miR-218-5p in sEV from dermal papilla cells grown as spheroids, which was associated with improved hair regeneration *in vivo* compared to ML-derived sEV (Hu et al., 2020).

For all these reasons, the production of sEV from cells cultured as spheroids represents a promising avenue for manufacturing sEV-based therapies. However, to date, most studies on spheroid-derived sEV have been conducted under static conditions, such as hanging drops or low-attachment plates. Few studies have investigated the characteristics of sEV derived from spheroids produced in STBr, despite this being critical for scaling up the process and maintaining a microenvironment conducive to cell viability and function. On the one hand, insufficient agitation can fail to keep spheroids or even cells in suspension, concomitant with long mixing times and the formation of substrate or pH gradients. On the other hand, excessive agitation can cause shear stress, potentially modifying the phenotype of the parental cells and, consequently, the characteristics of the produced EV (Colao et al., 2018). Moreover, the scalability of this production method remains largely unexplored. While some parameters, such as medium composition, seeding density, and controlled physicochemical factors, are independent of culture scale, hydrodynamics and mass transport conditions, like agitation and gas-sparging rates, must be adjusted to ensure a consistent cell microenvironment across different scales. Maintaining constant macroscopic parameters that describe the hydrodynamics of the system, such as volumetric power input (*P/V*) or impeller tip speed (*υ*_*tip*_) are common strategies used to stabilize the hydrodynamic microenvironment of cells across scales.

EV from pancreatic β cells may constitute promising tools for immunotherapy of type 1 diabetes, as they convey a cocktail of β antigens (Cianciaruso et al., 2017; Hasilo et al., 2017) and play a role in β cell homeostasis in physiological conditions (Chidester et al., 2020). Notably, in a mouse model of type 1 diabetes, the injection of β cell-derived EV reduces hyperglycemia and extends animal survival (Sun et al., 2019). These findings are associated with a decrease in macrophage infiltration and an increase in the vascularization of the islets of Langerhans. Interestingly, spheroid culture enhances β cell maturation and function (Hauge-Evans et al., 1999; Lock et al., 2011; Ntamo et al., 2021). We previously demonstrated the feasibility of producing β cell-derived sEV from mouse MIN6 cells cultured as spheroid in SpF (de Beaurepaire et al., 2024). However, to anticipate clinical demands, the process still faces the challenge of defining the criteria for scaling-up the process. Therefore, in this study, we aimed to establish a standardized and scalable spheroid culture in serum-free conditions for the production of sEV from a human β cell line. The 1.4E7 human β cell line derived from the electrofusion of a primary culture of human pancreatic islets with PANC-1, a human pancreatic ductal carcinoma cell line was selected (McCluskey et al., 2011). 1.4E7 cells were cultured in SpF at a constant working volume and agitation rate, with or without serum, to characterize the kinetics of cell growth, cell death, metabolism, and spheroid formation under quantified hydrodynamics conditions. Next, to determine the optimal agitation rate for a further scale-up, we evaluated whether the best strategy for standardizing spheroid formation was to keep either the *P/V* or the *υ*_*tip*_ constant in SpF. The SpF process was then transferred to a fully controlled and miniaturized bioreactor (Ambr^®^250) to assess the impact of each culture mode (ML *vs.* spheroids) and system (T-flask *vs.* SpF *vs.* STBr) on parental cell performance for the production of sEV, both quantitatively and qualitatively.

## 2 Materials and methods

### 2.1 Cell culture

The 1.4E7 human β cell line (ECACC) was cultured either in ML or as spheroids in RPMI-1640 medium supplemented with 10% fetal bovine serum (FBS) and 2 mM L-glutamine (all from Biosera), referred to as complete medium. Cultures were maintained in a humidified incubator at 37°C with 5% CO_2_. For ML culture, 1.4E7 cells were seeded at 2 × 10^4^ cells/cm^2^ in T-flasks with 0.2 mL/cm^2^ of complete medium and routinely passaged upon reaching 80%–90% confluence. For spheroid culture, 1.4E7 ML cultures were detached using trypsin and seeded at 0.3 × 10^6^ cells/mL in 0.125 L or 0.5 L SpF (Corning) in 80% of working volume. The SpF were maintained under clockwise agitation on a magnetic stir plate (2mag bioMIX) set at 90 rpm (0.125 L SpF) or 75 rpm (0.5 L SpF), resulting in a constant *P/V* of ∼8.5 W/m^3^. 1.4E7 spheroids were cultured either in complete medium or in a chemically defined medium (RPMI-1640 Advanced, Capricorn Scientific # RPMI-ADV-500ML). This medium which contains insulin, transferrin and bovine serum albumin was supplemented with 4 mM L-glutamine, 100 IU/mL penicillin, and 100 µg/mL streptomycin (Eurobio), and referred to as production medium. Cultures were regularly monitored for *mycoplasma* contamination using the MycoAlert™ detection kit (Lonza). The maximal growth rate *µ*_*max*_ (h^−1^) was determined as the slope of the linear regression of the natural logarithm transformation of the exponential growth phase. Population doubling time (*PDT*, h) was inferred as *PDT = ln(2)/µ*_*max*_.

### 2.2 Ambr^®^250 stirred-tank bioreactor operation

1.4E7 spheroids were cultured in the Ambr^®^250 modular STBr using dual 30° three-segment pitched-blade impeller mammalian culture vessels (Sartorius Stedim). The cells were inoculated at 0.3 × 10^6^ cells/mL in 245 mL of production medium. The temperature was set to 37°C, with an agitation rate of 300 rpm (to achieve ∼8.5 W/m^3^), pH maintained at 7, and dissolved oxygen (DO) saturation set to 50%. pH was regulated by the addition of 1M NaOH and sparged CO_2_, while DO was maintained through air sparging at a maximum rate of 2.45 mL/min.

### 2.3 Spheroids count and size measurement

Samples from SpF or STBr cultures were collected, and 30–200 µL was distributed in triplicate into a flat-bottomed 96-well plate for spheroid counting. Images were captured from each well using an Axiovert A1 FL LED Inverted Microscope coupled with an AxioCam MRc (Zeiss), and the largest diameter of each spheroid was measured using Fiji software. The interfacial area *a* (mm^2^/mL) of either the spheroids or the free cells (i.e., cells not incorporated into spheroids) in 3D cultures was calculated using Equation 1 where *r* is the mean radius of the free cells or spheroids (in mm), measured using Fiji software, and [*X*] is the concentration of spheroids or free cells (per mL).

$$a=4\times \pi \times r^{2}\times \left[X\right]$$ (1)

### 2.4 Hydrodynamic characterization

Mixing and hydrodynamics in SpF and STBr cultures were characterized by various parameters. The Reynolds number (*Re*, dimensionless) was calculated using Equation 2, while the laminar-turbulent transition (*Re*_*T*_) was estimated using Equation 3 (Grenville and Nienow, 2003). The volumetric power input *P/V* (W/m^3^) was calculated using Equation 4, the mixing time *Θ*_*m*_ (s) was estimated using Equation 5, the mean shear rate $\langle \dot{ϒ}\rangle$ (s^−1^) using Equation 6, the mean Kolmogorov length scale $\langle \lambda_{K}\rangle$ (µm) using Equation 7 and the impeller tip speed *υ*_*tip*_ (m/s) using Equation 8.

$$Re=\frac{\;\rho \times N\times D^{2}\;}{µ}$$ (2)

$${Re}_{T}=6370\times {N_{P}}^{‐1/3}$$ (3)

$$P/V=\frac{N_{p}\times \rho \times N^{3}\times D^{5}\;}{V}$$ (4)

$$\Theta_{m\;}=5.9\times {\langle \varepsilon \rangle }^{-1/3}\times {\left(\frac{D}{T}\right)}^{-1/3}\times T^{\;2/3}\text{ with }\langle \varepsilon \rangle =P/\left(\rho \times V\right)$$ (5)

$$\dot{\langle \gamma \rangle }={\left(\frac{P}{µ\times V}\;\right)}^{1/2}$$ (6)

$$\langle \lambda_{K}\rangle ={\;\left(\frac{\mu^{3}}{\rho^{3}\times \langle \varepsilon \rangle }\right)}^{1/4}$$ (7)

$$v_{tip}=\pi \times N\times D$$ (8)

In these equations, *N* (s^−1^) is the agitation rate, *D (m) is* the diameter of the impeller, *ρ* is the density of the culture medium (assumed to be that of water at 37°C = 993 kg/m^3^) and μ is the dynamic viscosity (assumed to be that of water at 37°C = 7 × 10^−4^ Pa·s). For *N*_*P*_ (dimensionless), we used the experimentally determined values reported by Rotondi et al*.*: 2.51 for a single flat paddle impeller, mimicking SpF, and 1.37 for the dual 30° three-segment pitched-blade impeller of the Ambr^®^250 STBr (Rotondi et al., 2021). *V (*m^3^) is the volume of the medium; *T (m)* is the vessel diameter, and $\langle \varepsilon \rangle$ (W/kg) is the mean specific energy dissipation rate. A summary of the geometric characteristics of the SpF and the STBr, along with the calculated and fixed mixing and hydrodynamic values for this study, are presented in Table 1.

**TABLE 1:** Summary of the geometric, mixing and hydrodynamic characteristics of the 0.125 L and 0.5 L SpF, and the Ambr^®^250 STBr.

Characteristic	0.125 L SpF	0.5 L SpF	Ambr^®^250 STBr
Vessel diameter, *T* (m)	0.07	0.1	0.061
Impeller diameter, *D* (m)	0.04	0.059	0.026
Volume, *V* (m^3^)	1 × 10^−4^	4 × 10^−4^	2.45 × 10^−4^
Liquid height, *H* (m)	0.035	0.064	0.106
Geometric ratio, *H/T*	0.5	0.64	1.74
Geometric ratio, *D/T*	0.57	0.59	0.43
Agitation rate, *N* (rpm)	90	75	300
Power number, *N*_*P*_ (−)	2.51	2.51	1.37
Reynolds number, *Re* (−)	3,405	6,173	4,795
Laminar–turbulent transition, *Re*_*T*_ (−)	4,687	4,687	5,735
Volumetric power input, *P/V* (W/m^3^)	8.6	8.7	8.2
Mean specific energy dissipation rate, $\langle \varepsilon \rangle$ (W/kg)	8.7 × 10^−3^	8.8 × 10^−3^	8.3 × 10^−3^
Mixing time, *Θ*_*m*_ (s)	5.9	7.4	6.0
Shear rate, $\langle \dot{\gamma }\rangle$ (s^-1^)	111	111.5	108.5
Mean Kolmogorov lenght scale, $\langle \lambda_{K}\rangle$ (µm)	79.7	79.5	80.6
Impeller tip speed, *υ*_*tip*_ (m/s)	0.19	0.23	0.41

### 2.5 Viability assessment

Cell viability was assessed using two distinct methods: trypan blue exclusion and estimation of dead and lysed cells through lactate dehydrogenase (LDH) activity in the cell culture supernatant. Specifically for spheroid cultures in SpF or STBr, samples were collected and centrifuged at 300 g for 5 min; then the pellets were dissociated with trypsin to allow for viable and dead cell count via trypan blue exclusion. In parallel, LDH activity in cell culture supernatants was measured as an indicator of cell damage and death (Legrand et al., 1992) with the Cytotoxicity Detection Kit (Roche) with rabbit muscle L-LDH as a standard (#10127230001, Roche). Absorbance was measured using the FLUOstar OPTIMA plate reader (BMG Labtech). In parallel, 1.4E7 cells grown as ML or spheroids in complete medium were lysed with 2% Triton X-100 to assess intracellular LDH levels, this being used to determine the number of dead and lysed cells in the culture supernatant. Lastly, cell viability was calculated using Equation 9 where [*X*]_*alive*_ is the viable cell concentration determined using trypan blue counting and [*X*]_*dead*_ is the estimated lysed cell concentration in cell culture supernatant.

$$Viability\;\left(\%\right)=\frac{{\left[X\right]}_{alive}}{{\left[X\right]}_{alive}+{\left[X\right]}_{dead}}\times 100$$ (9)

### 2.6 Analysis of metabolites in cell culture medium

Cell culture medium was collected, centrifuged at 300 g for 10 min, and stored at −80°C until metabolites were analyzed. The concentrations of glucose, glutamine, lactate and ammonium were quantified using the Gallery™ analyzer (ThermoFisher Scientific). Substrate (*S*) specific consumption rates (*q*_*S*_) for glucose and glutamine, and product (*Pr*) specific production rates (*q*_*P*_) for lactate and ammonium, between *i*-1 and *i* samplings (spaced by *Δt* interval), were determined respectively by Equations 10, 11

$$q_{S}=\frac{-1}{{\left[X\right]}_{i}}\times \frac{{\left[S\right]}_{i}-{\;\left[S\right]}_{i‐1}}{\Delta t}$$ (10)

$$q_{P}=\frac{1}{{\left[X\right]}_{i}}\times \frac{{\left[\mathrm{Pr}\right]}_{i}-{\;\left[\mathrm{Pr}\right]}_{i‐1}}{\Delta t}$$ (11)

Lactate-to-glucose and ammonium-to-glutamine metabolic yields (*Y*_*Lac/Glc*_ and *Y*_*NH4/Gln*_, respectively), as well as cell-to-glucose and cell-to-glutamine growth yields (*Y*_*cells/Glc*_ and *Y*_*cells/Gln*_, respectively), were determined as the slope of the linear regressions of respectively f([S]_i0_-[S]_i_) = [Pr]_i_-[Pr]_i0_; and f([X]_i_-[X]_i0_) = [S]_i0_-[S]_i_, between 0 and 72 h.

### 2.7 1.4E7-derived sEV production

All relevant data from our experiments were submitted to the EV-TRACK knowledgebase (EV-TRACK ID: EV240160) (Van Deun et al., 2017). Cells were expanded through 12 to 18 passages to produce sEV, reaching a cumulative population doubling level (*PDL*, Equation 12) of 18.8–33.4. *X*_*harvested*_ corresponds to the total number of viable cells harvested at each passage, and *X*_*seeded*_ corresponds to the total number of seeded cells.

$$PDL=\frac{\ln\left(X_{harvested}\right)\;‐\;\ln\left(X_{seeded}\right)\;}{\ln\left(2\right)}$$ (12)

For ML cultures, after 72 h of culture (∼80% confluence), cells were washed twice with PBS, and the complete medium was replaced with 0.12 mL/cm^2^ of production medium for 24 h. For 3D cultures in SpF and STBr, both spheroid formation and sEV production were performed in the production medium. After 48 h of culture, 1.4E7 spheroids were allowed to settle, and the culture medium was replaced with a fresh production medium (∼85% renewal).

For each sEV batch, around 400 mL of conditioned medium was used (Supplementary Table 1), obtained from 10 to 19 flasks for ML, two vessels for STBr, or single 0.5L SpF.

### 2.8 1.4E7-derived sEV isolation

After a 24 h sEV production period, 1.4E7-derived sEV were isolated using a method combining differential centrifugation, tangential flow filtration (TFF), ultrafiltration (UF), and size exclusion chromatography (SEC). Briefly, the production medium was collected and sequentially centrifuged to remove cell debris and large extracellular vesicles: 300 g for 10 min, 2,000 g for 20 min, and 16,000 g for 20 min, followed by filtration through a 0.22 µm PES filter. sEV were isolated from the conditioned medium by five cycles of diafiltration in PBS using a 300 kDa MidiKros column (Repligen) on a KrosFlo Research IIi TFF instrument (Spectrum Europe). The input flow rate was set to 50 mL/min, with transmembrane pressure regulated below 300 mbar. Twenty mL of retentat were recovered in PBS and further concentrated down to 150 µL using a 100 kDa AMICON (Merck Millipore) filter by centrifugation at 2,000 g for 20 min. sEV were then passed through a qEV Gen2 35 nm single SEC column (Izon). sEV were collected in flow-through fractions two, three, and four in PBS filtered 0.1 µm. sEV were stored at −80°C in PBS containing 25 mM trehalose (Sigma-Aldrich) (Bosch et al., 2016).

### 2.9 Nanoparticle tracking analysis

sEV size and concentration were determined by nanoparticle tracking analysis of the tetraspanin-labeled particles using the ZetaView^®^ PMX 220 TWIN (Particle Metrix) and the associated software (ZetaView 8.05.16). Briefly, sEV were incubated with AF-488-conjugated anti-human CD81 (454720, Bio-Techne), anti-human CD9 (209306, Bio-Techne), and anti-human CD63 (H5C6, BioLegend) antibodies at a 1:200 dilution overnight at 4°C. After incubation, samples were diluted in 0.1 µm-filtered PBS to achieve a minimum 1:50,000 final dilution of the antibodies. Before analysis, the ZetaView^®^ was calibrated using 100 nm polystyrene standard particles. For acquisition, samples were excited with a 488 nm laser coupled with a 500 nm filter. Videos were recorded at 25°C across 11 positions, with a sensitivity of 95, a shutter speed of 100, and a frame rate of 30 frames per second.

### 2.10 Protein quantification

For total protein analysis, cells were lysed in RIPA lysis buffer (ThermoFisher Scientific) containing 1% proteases/phosphatases inhibitors (Ozyme). sEV were lysed in 0.1% Triton X-100 (Sigma-Aldrich). Protein concentration was determined using Coomassie plus assay reagent (ThermoFisher Scientific) following the manufacturer’s recommendations. Protein absorbance was read on the FLUOstar OPTIMA plate reader.

### 2.11 Cryo-electron microscopy

For morphological analysis, sEV were prepared for cryo-electron microscopy as described previously (Giri et al., 2020) and imaged on a Tecnai G2T20 Sphera electron microscope (FEI Company) equipped with a CMOS camera (XF416, TVIPS) at 200 kV. Micrographs were acquired under low electron doses, using the camera in binning mode 1, at a nominal magnification of ×50,000.

### 2.12 Western blot

50 µg of cellular protein lysate or 5 × 10^9^ sEV were denatured in Bolt™ sample buffer (Thermo Fisher Scientific) and separated on a 4%–12% Bis-Tris Plus gradient SDS-PAGE gel under non-reducing conditions, followed by transfer to a nitrocellulose membrane (Thermo Fisher Scientific). Membranes were blocked with TBS containing 0.05% Tween-20% and 5% milk, then incubated with the following primary antibodies from BioLegend: mouse anti-CD63 (H5C6, 1:1,000), mouse anti-CD81 (5A6, 1:500), mouse anti-CD9 (HI9a, 1:1,000), mouse anti-LAMP1 (H4A3, 1:500), HRP-rat anti-Flotillin-1 (W16108A, 1:2,500), HRP-rat anti-β-actin (W16197A, 1:10,000), mouse anti-Hsp70 (W27, 1:1,000), or rat anti-Calnexin (W17077C, 1:1,000). Membranes were then incubated with the appropriate horseradish peroxidase-conjugated secondary antibodies: polyclonal rabbit anti-mouse (1:2,000, Agilent Technologies) or polyclonal goat anti-rat (1:20,000, Thermo Fisher Scientific), except for β-actin and Flotillin-1 antibodies. Signals were detected using enhanced chemiluminescence substrate Pico or Femto (Thermo Fisher Scientific), depending on the target, and visualized on a Fusion FX6 instrument (Thermo Fisher Scientific).

### 2.13 sEV analyzes with ExoView™

sEV were analyzed using the ExoView™ Human Tetraspanin Kit (Unchained Labs) according to the manufacturer’s instructions. Briefly, sEV were diluted to a final concentration of 2 × 10^8^ sEV/mL in the provided incubation solution. A volume of 50 µL of the sEV solution (1 × 10^7^ sEV) was added to silicon dioxide chips coated with antibodies against human CD9 (HI9a), CD63 (H5C6), and CD81 (JS-81). The chips were incubated at room temperature for 16 h and then washed with the supplied washing solution. Subsequently, the chips were incubated for 1 h at room temperature with fluorochrome-conjugated antibodies against CD9 (1:500, HI9a, CF^®^488A), CD63 (1:500, H5C6, CF^®^647), and CD81 (1:500, JS-81, CF^®^-555), prepared in the supplier’s blocking solution. After incubation, the chips were washed, dried, and imaged using the ExoView™ R100 platform and the ExoView Scanner 3.2.2 acquisition system. Data were analyzed using ExoView Analyzer 3.2 software. The data from each tetraspanin capture spot (CD9, CD63, and CD81) were calculated by subtracting the IgG control spot values.

### 2.14 Confocal imaging of the cellular localization of tetraspanins

For immunofluorescence staining, 3 × 10^4^ 1.4E7 cells/cm^2^ were seeded onto 8-chamber Nunc LabTek slides. The following day, cells were washed in PBS and fixed with PBS 4% paraformaldehyde for 15 min at room temperature. Cells were rehydrated in PBS and permeabilized in PBS containing 0.2% Tween-20% and 5% BSA for 30 min at room temperature. Cells were washed in PBS and incubated in PBS 5% BSA blocking buffer for 30 min at room temperature, followed by overnight incubation at 4°C with primary antibodies or isotypic controls diluted in blocking buffer [anti-human CD63, H5C6, 1:50 (BioLegend); anti-human CD81, 454720, 1:25 (Bio-Techne); anti-human CD9, 209306, 1:25 (Bio-Techne)]. The next day, cells were washed and incubated with secondary AF488-anti-mouse IgG H+L antibody (Thermo Fisher Scientific, #A32723) for 1 h at room temperature. Nuclei were counterstained with Hoechst 33342 (Sigma-Aldrich) at 1 μg/mL for 10 min at 37°C before imaging on an LSM780 confocal microscope (Zeiss) with a Plan-Apochromat objective lens 63x/1,4 Oil DIC M27, and analysis with Fiji software (Schindelin et al., 2012).

### 2.15 Statistical analyzes

Statistical tests were performed using GraphPad Prism 8. Data are presented as means ± SD. Differences between two groups only were analyzed using the Mann-Whitney test. The Kruskal–Wallis test, followed by Dunn’s multiple comparisons test, was used for comparing data sets of three or more groups. Correlation analyzes were performed using Spearman’s rank correlation test. Significant differences are indicated in the figures as: *p < 0.05; **p < 0.01; ***p < 0.001; ****p < 0.0001.

## 3 Results

### 3.1 Impact of transitioning from a static 2D culture to a stirred-3D culture system on cell growth, death, metabolism and spheroids formation kinetics

The human β cell line 1.4E7 was cultured either in T-flasks as 2D static ML with a complete medium containing 10% FBS, or in suspension spheroids in 0.125 L SpF at an agitation rate of 90 rpm and a seeding density of 0.3 × 10^6^ cells/mL. The 3D cultures were maintained in either a complete medium; or in a chemically defined medium, without FBS (production medium), to implement a fully serum-free 3D culture process and prevent contamination from serum-derived EV.

**FIGURE 1:**
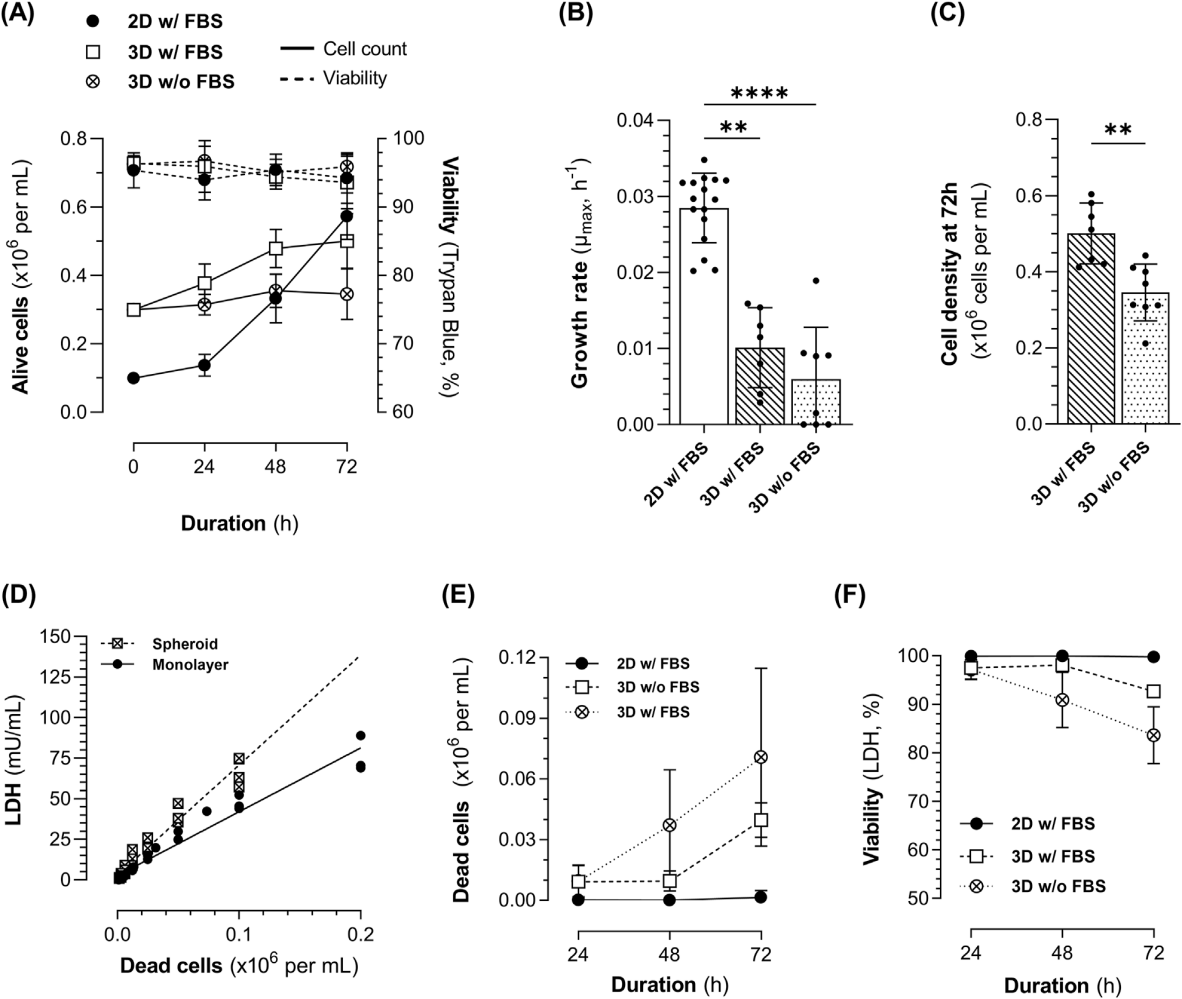
Kinetics of 1.4E7 cells cultured in 2D static T-flasks as monolayers (ML, black dot) in medium with FBS or in 3D in suspension in spinner flasks (SpF) in medium with (white square) or without FBS (white crossed dot). **(A)** Growth curves (continuous line) and viability determined using trypan blue exclusion (dashed line). **(B)** Growth rate and **(C)** cell density at 72 h. **(D)** Intracellular lactate dehydrogenase (LDH) content of 1.4E7 cells grown as ML (black dot) or spheroids (crossed square). **(E)** Evolution of dead cells in culture, determined from LDH measurements in the cell culture supernatants. **(F)** Viability was determined by assessing dead cells concentration based on LDH measurements in the cell culture supernatants. Results are expressed as means ± SD. Numbers of biological replicates from independent experiments were as follows: 2D (n = 16), 3D with FBS (n = 7), and 3D without FBS (n = 8). Statistical analyzes were performed using the Kruskal–Wallis test with Dunn’s multiple comparisons test for three-group comparisons, and the Mann-Whitney test for two-group comparisons (**p < 0.01; ****p < 0.0001).

Regardless of FBS supplementation, 3D spheroid cultures maintained high cell viability (>93%), as assessed using trypan blue exclusion, throughout the process, with no significant differences between the ML and 3D cultures (Figure 1A). However, 3D spheroid cultures significantly reduced 1.4E7 cell growth compared to 2D static ML culture (Figure 1B). The average growth rate (*µ*_*max*_) in ML culture was 0.028 h^−1^, compared to 0.01 h^−1^ in 3D cultures with 10% FBS, and 0.006 h^−1^ in 3D cultures without FBS, corresponding to *PDT* of 25.1 h ± 4.6, 100.2 h ± 76.2, and 145.1 h ± 178, respectively. Although the *µ*_*max*_ of cells grown as spheroids with or without FBS did not significantly differ, fewer cells were obtained after 72 h of culture in the absence of FBS (Figure 1C).

LDH activity was assessed in cell culture supernatants as an indicator of cell death. 1.4E7 cells, grown as ML or spheroids, were first lysed in 2% Triton X-100 to evaluate their LDH content. Intracellular LDH levels were notably higher in the lysates of 1.4E7 spheroids compared to ML (Figure 1D). This allowed the estimation of the number of dead and lysed cells based on LDH activity in the supernatant depending on culture conditions (Figure 1E). Since LDH activity in ML culture supernatants was minimal or undetectable, higher cell viability was inferred using this method, compared to 3D cultures (Figure 1F, ML *vs*. 3D with FBS: p < 0.05; ML *vs.* 3D without FBS: p < 0.0001). At 72 h, 3D cultures exhibited a cell viability of 92.7% ± 1.1 in the presence of FBS compared to 83.6% ± 5.8 in its absence, which was not significantly different. Thus, LDH measurement is a sensitive method for 3D cultures, allowing the detection of lysed cells that are not identified using trypan blue exclusion.

**FIGURE 2:**
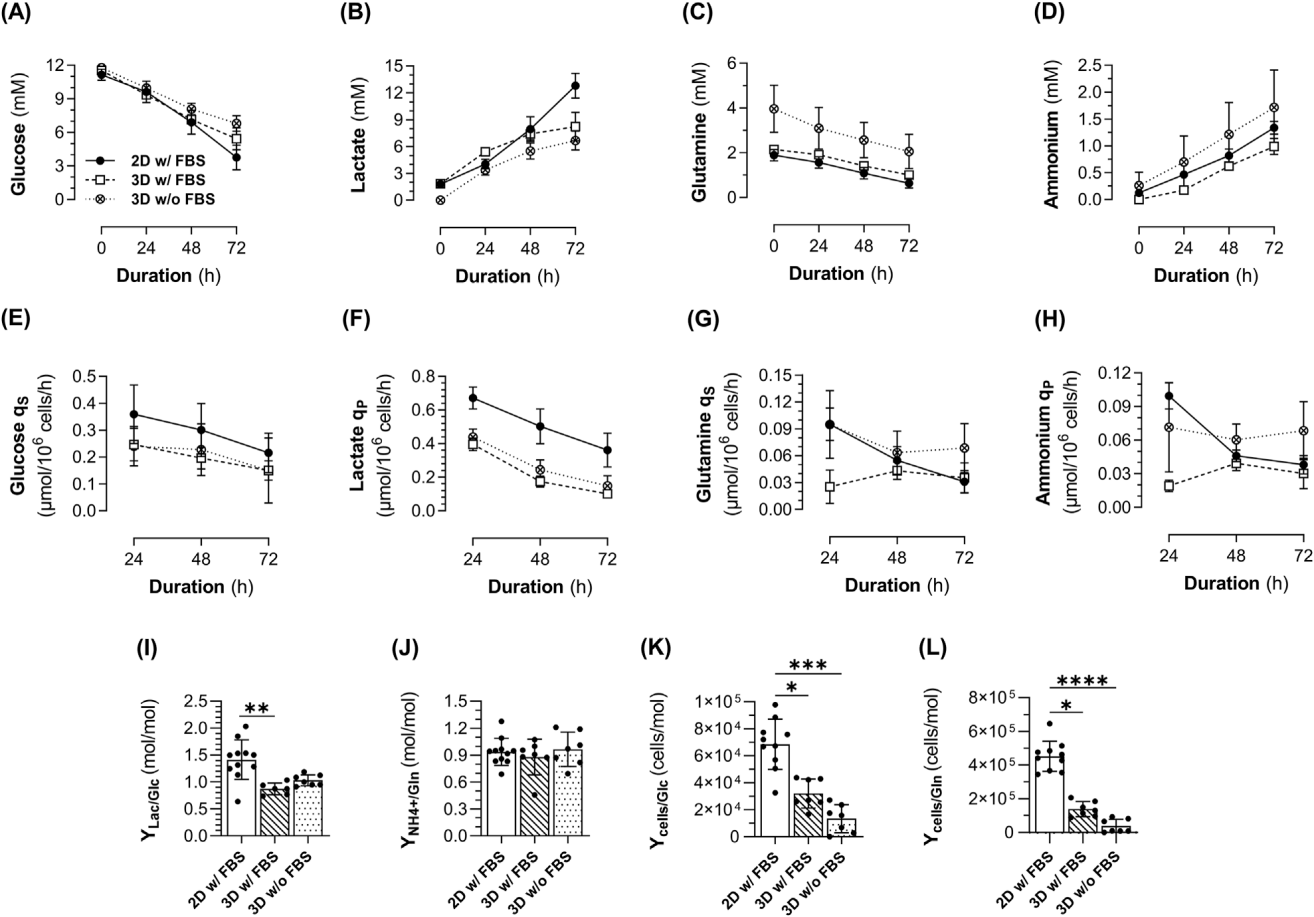
Metabolite profile of 1.4E7 cells cultured in 2D static T-flasks as monolayers (ML, black dot) in medium with FBS or in 3D in suspension in spinner flasks (SpF) in medium with (white square) or without FBS (white crossed dot). **(A)** Glucose, **(B)** lactate, **(C)** glutamine and **(D)** ammonium concentrations measured in the cell culture supernatant. **(E)** Glucose and **(G)** glutamine specific consumption rates (*q*_*S*_), **(F)** lactate and **(H)** ammonium specific production rates (*q*_*P*_). **(I)** Lactate-to-glucose and **(J)** ammonium-to-glutamine metabolic yields (*Y*_*Lac/Glc*_ and *Y*_*NH4/Gln*_, respectively). **(K)** Cell-to-glucose and **(L)** cell-to-glutamine growth yields (*Y*_*cells/Glc*_ and *Y*_*cells/Gln*_, respectively). Results are expressed as means ± SD. Numbers of biological replicates from independent experiments were as follows: 2D (n = 11), 3D with FBS (n = 7), and 3D without FBS (n = 7). Statistical analyzes were performed using the Kruskal–Wallis test with Dunn’s multiple comparisons (*p < 0.05; **p < 0.01; ***p < 0.001; ****p < 0.0001).

Monitoring the metabolites in the culture medium confirmed the absence of substrate limitation (glucose and glutamine, Figure 2A, C) and excess metabolic waste (lactate <20 mM and ammonium ions <5 mM, Figure 2B, D) (Hassell et al., 1991; Cruz et al., 2000). The glucose *q*_*S*_ (Figure 2E) and lactate *q*_*P*_ (Figure 2F) decreased over the culture duration in all conditions. However, both parameters were higher in ML cultures compared to 3D cultures, regardless of FBS supplementation, which is consistent with higher cell proliferation in ML cultures. Interestingly, the glutamine *q*_*S*_ (Figure 2G) and ammonium *q*_*P*_ (Figure 2H) gradually decreased with time in ML cultures, in line with exponential growth, which was not the case in 3D cultures. A higher lactate-to-glucose yield (*Y*_*Lac/Glc*_) was observed in ML cultures (1.4 mol/mol ±0.4) compared to 3D cultures (0.9 mol/mol ±0.1 in the presence of FBS and 1 mol/mol ±0.1 in the absence of FBS) (Figure 2I). This relatively high ratio in ML cultures suggests that cells are primarily using glycolysis, a phenomenon commonly seen under hypoxia, but also in proliferating cells regardless of oxygen concentration (known as Warburg effect) (Vander Heiden et al., 2009). In contrast, the ratio of 1 mol/mol in 3D cultures indicates a lower lactate production relative to glucose consumption, suggesting that cell metabolism shifted to oxidative phosphorylation which allows for more efficient ATP production from glucose (∼36 ATP instead of 2). Concerning the ammonium-to-glutamine metabolic yield (*Y*_*NH4/Gln*_) (Figure 2J), a similar ratio of <1 mol/mol was observed in all conditions, suggesting a balanced metabolic state where ammonium production is proportional to glutamine availability, indicating efficient glutamine utilization. However, the growth yield, expressed as cells generated per mole of glucose (*Y*_*cells/Glc*_, Figure 2K) or glutamine (*Y*_*cells/Gln*_, Figure 2L) consumed, was higher in ML cultures, indicating a more efficient utilization of these metabolites for cell growth compared to 3D cultures.

**FIGURE 3:**
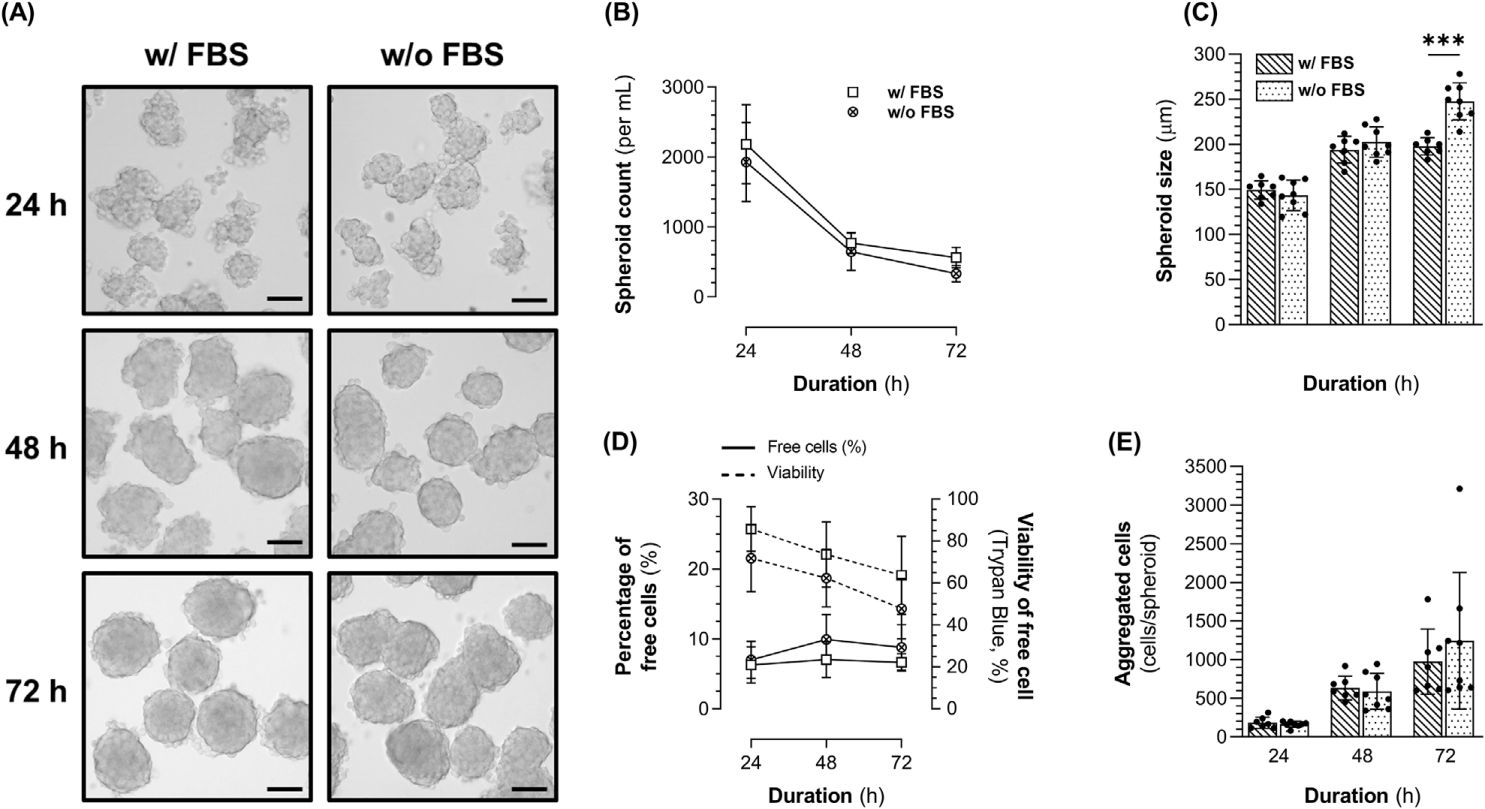
Characteristics of 1.4E7 spheroids generated in 0.125 L spinner flasks set at 90 rpm in complete medium containing 10% FBS or in production medium in the absence of FBS. **(A)** Representative images of the 1.4E7 spheroids at 24 h, 48 h, and 72 h. Scale bar = 100 µm. Evolution of the spheroids **(B)** concentration and **(C)** size. **(D)** Evolution of the free cell percentage (continuous line in spheroid) cultures and their viability determined using trypan blue exclusion (dashed line). **(E)** Number of cells per spheroid across the culture duration. Results are expressed as means ± SD. Numbers of biological replicates from independent experiments were as follows: 3D with FBS (n = 7) and 3D without FBS (n = 8). Statistical analyzes were performed using the Mann-Whitney test (***p < 0.001).

In 3D cultures, 1.4E7 cells rapidly self-agglomerated until forming well-defined and spherical spheroids regardless of FBS concentration (Figure 3A). The concentration of spheroids decreased with time (Figure 3B), while their size increased (Figure 3C) suggesting that spheroids grew larger as they fused together. While after 48 h, we observed similar spheroids’ concentration and size with or without FBS, at 72 h larger but fewer spheroids were obtained in the absence of FBS. Indeed, we counted 333 spheroids ±116 per mL, with a size of 248 µm ± 20 in the absence of FBS against 562 spheroids ±146 per mL (p < 0.01), with a size of 198 µm ± 10 (p < 0.001) in the presence of FBS. Six to 10% of the cells remained as alive or dead free cells (*i.e.* not incorporated within spheroids), and their viability decreased over time in culture (Figure 3D), suggesting that these cells likely underwent anoïkis due to the lack of adhesion. Interestingly, free cell viability appeared lower in the absence of FBS, although the differences were not statistically significant at any time point. Lastly, similar numbers of cells per spheroid were obtained regardless of FBS supplementation (Figure 3E). In the absence of FBS, spheroids contained 590 cells ±232 at 48 h, increasing to 1244 cells ±883 at 72 h.

In conclusion, while 2D ML cultures achieved higher growth yields, 3D spheroid cultures allowed to culture 1.4E7 cells in suspension while maintaining high cell viability. Although the proliferative capacity of cells was lower in 3D cultures, the cells metabolism shift towards oxidative pathway respiration improving glucose utilization for ATP production. Additionally, 3D cultures of 1.4E7 cells in a serum-free and chemically defined medium preserved equivalent cell viability, metabolic profiles, and spheroid morphology while avoiding contamination of the medium by exogenous EV.

For further investigation of the upstream culture mode on 1.4E7 spheroids and sEV derived thereof, we used an sEV production process comprising an expansion phase in 2D ML cultures containing FBS, followed by a transition to a 3D culture system using a serum-free, chemically defined medium, where spheroids were formed for 48 h. Subsequently, the medium was renewed for an additional 24 h culture phase to produce sEV.

### 3.2 Hydrodynamic characterization of 3D cultures in SpF and scale-up strategies assessment

**FIGURE 4:**
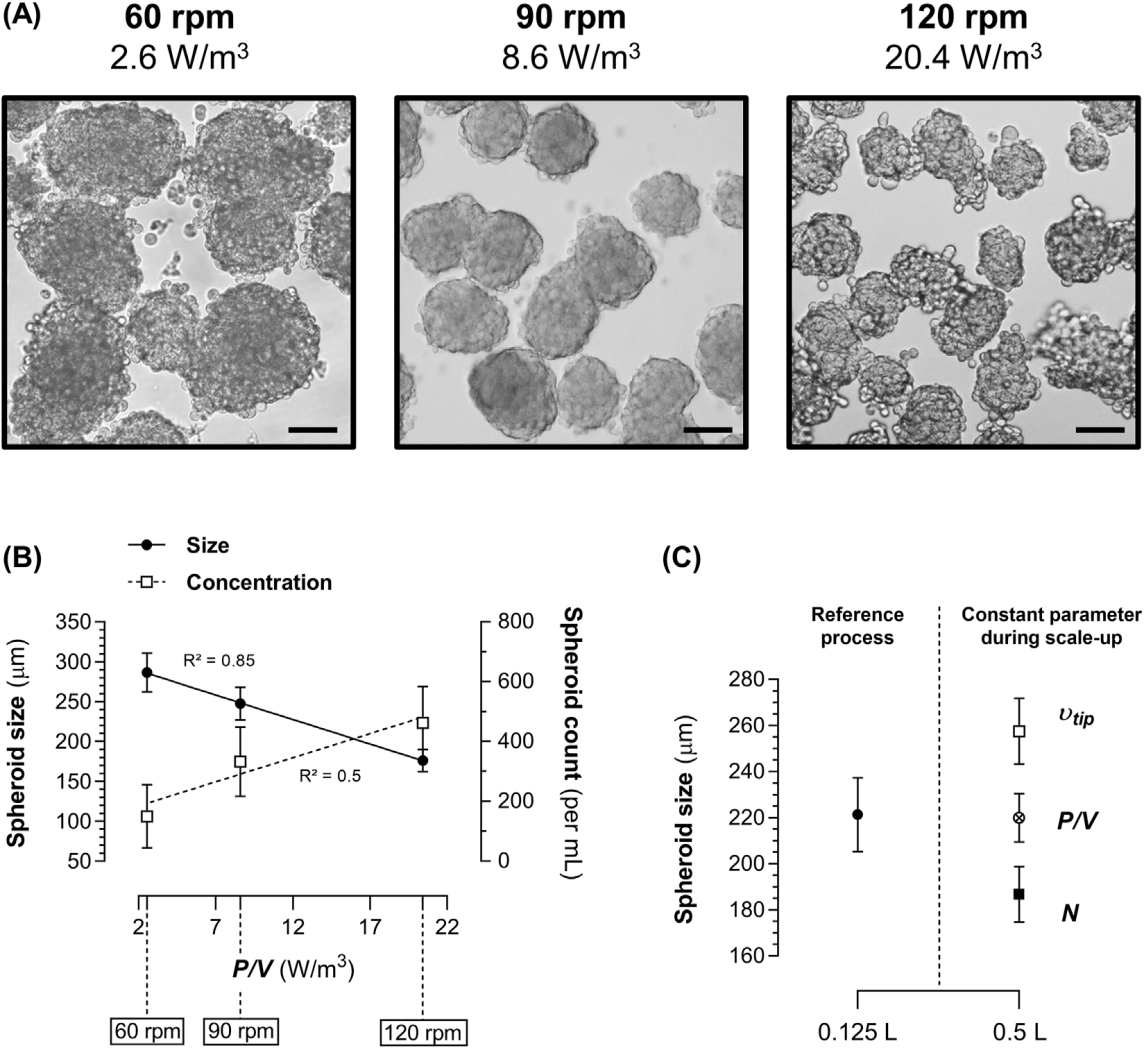
Impact of hydrodynamics and scale-up strategies on spheroid formation in spinner flasks (SpF) in production medium. **(A)** Representative images of the 1.4E7 spheroids obtained after 72 h in a 0.125 L SpF at agitation rates (*N*) of 60 rpm (2.6 W/m^3^), 90 rpm (8.5 W/m^3^), and 120 rpm (20.5 W/m^3^). Scale bar = 100 µm. **(B)** Correlation between the *P/V* and the size (continuous line) or the concentration (dashed line) of 1.4E7 spheroids in a 0.125 L SpF after 72 h of culture. **(C)** Size of 1.4E7 spheroids after 72 h of culture in a 0.125 L SpF at 90 rpm compared to those generated in a 0.5 L SpF at a constant *N* (90 rpm, black square), at a constant *P/V* of 8.6 W/m^3^ (75 rpm, crossed dot), or at a constant impeller tip speed (*υ*_*tip*_) of 0.19 m/s (61 rpm, white square). Each culture was performed at 80% of the SpF working volume. Results are expressed as means ± SD. Numbers of biological replicates from independent experiments were as follows: **(A,B)**, 60 rpm (n = 5), 90 rpm (n = 8), 120 rpm (n = 6); - **(C)**, 0.125 L (n = 5), 0.5 L *υ*_*tip*_ (n = 3), 0.5 L *P/V* (n = 4), 0.5 L *N* (n = 3).

The impact of hydrodynamics on spheroid formation was assessed by measuring spheroid size and concentration after 72 h of culture at different agitation rates (60, 90, and 120 rpm) in 0.125 L SpF with a constant volume of production medium (100 mL) and, therefore, a constant liquid height (*H*) and liquid height-to-vessel diameter ratio (*H/T*). This resulted in *P/V* values of 2.6, 8.6, and 20.4 W/m^3^, respectively. Visually, 1.4E7 cells cultured at 60 rpm tended to form larger and more heterogeneous aggregates, while cultures at 120 rpm produced smaller aggregates with less-defined morphology. In contrast, cultures at 90 rpm consistently generated homogenous and well-defined spheroids (Figure 4A).

These agitation rates in 0.125 L SpF correspond to the operational window that enables spheroid formation. Below 60 rpm and above 120 rpm, large (several mm) heterogeneous clusters, consisting mainly of dead cells, along with a significant number of free cells, were obtained (data not shown).

Thus, within this operational window, we observed that *P/V* was linearly and negatively correlated to spheroid size (Spearman, R^2^ = 0.85, p < 0.0001) and positively to spheroid concentration (R^2^ = 0.5, p < 0.001) (Figure 4B). As spheroid formation was dependent on the system’s *P/V*, we applied a constant *P/V*-based strategy to standardize spheroid formation during process scale-up. As expected, in a larger SpF (0.5 L), applying a constant *P/V* of ∼8.6 W/m^3^ generated spheroids of similar size to those obtained in the reference 0.125 L SpF at 90 rpm (Figure 4C). Conversely, maintaining a constant agitation rate of 90 rpm in a 0.5 L SpF increased the *P/V* to 15 W/m^3^, resulting in smaller spheroids (p < 0.05). An alternative scale-up strategy, which uses a constant *υ*_*tip*,_ required setting the agitation rate to 61 rpm. This condition, with a lower *P/V* value of 4.7 W/m^3^, generated larger spheroids (p < 0.05). These data demonstrate that spheroid formation strongly depends on the system’s *P/V*, and maintaining this parameter constant during process scale-up in geometrically similar vessels is crucial to standardizing spheroid size and morphology.

### 3.3 Process transfer to STBr and 1.4E7 spheroid-derived sEV production

The SpF process was subsequently transferred to the fully controlled Ambr^®^250 STBr by applying a constant *P/V* of approximately 8.5 W/m^3^, despite significant geometric and impeller design differences between the SpF and the STBr. Additionally, in contrast to SpF, volumetric aeration was introduced through the sparger in STBr, allowing for oxygen level control. However, its impact on the overall P/V under our conditions was minimal and negligible. 1.4E7-derived sEV were produced in both stirred systems, following a process in two steps: (i) a spheroid formation phase of 48 h, followed by (ii) an ∼85% renewal of the production medium for an additional 24 h, before collection of the supernatants to isolate sEV.

**FIGURE 5:**
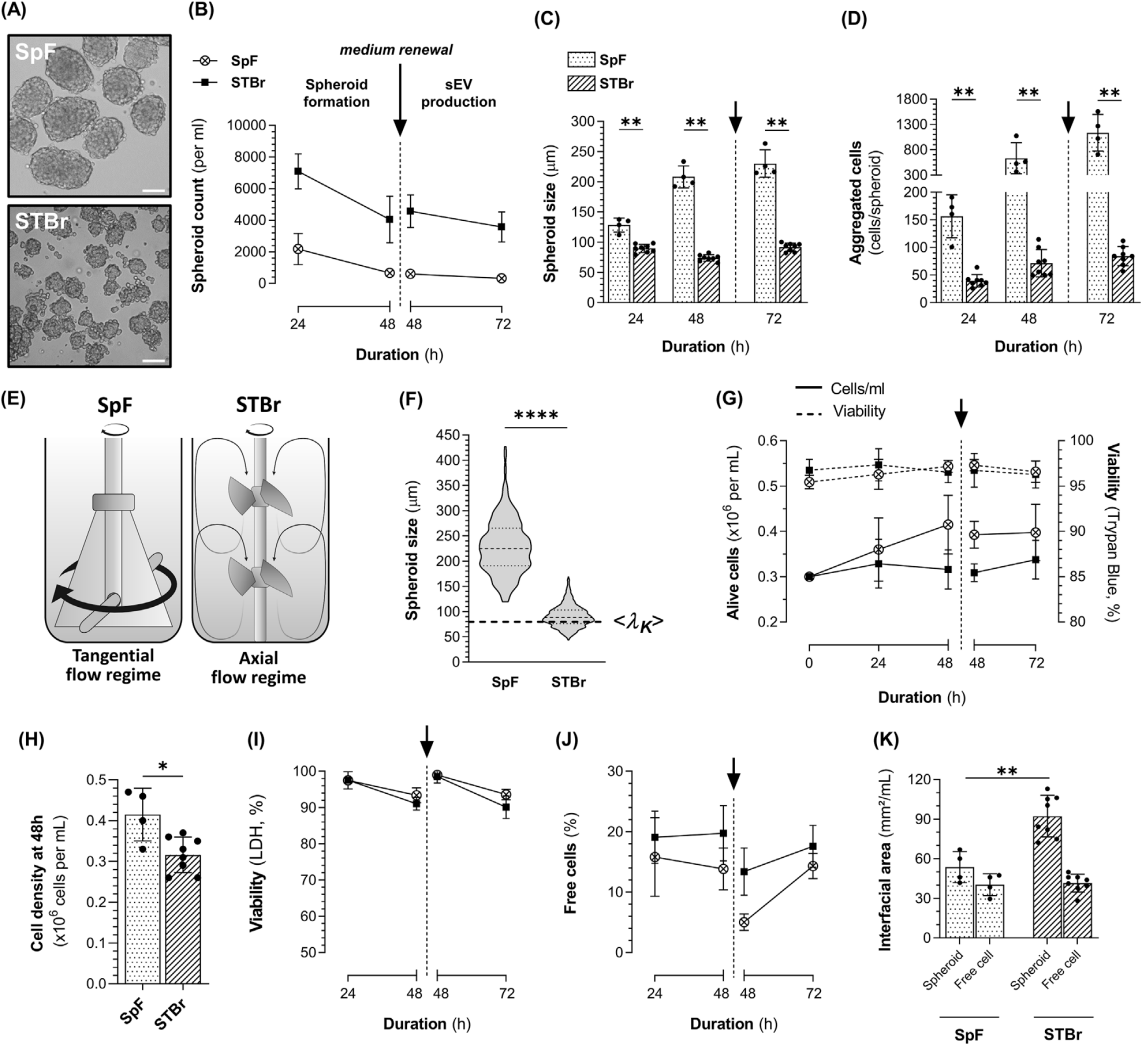
Kinetics of 1.4E7 cell 3D cultures in 0.5 L spinner flasks (SpF) or stirred-tank bioreactors (STBr) in production medium at a constant *P/V* of 8.5 W/m^3^. 1.4E7 cells were cultured in an initial volume of 430 mL in SpF or 245 mL in STBr to form spheroids. After 48 h, the production medium was renewed for a subsequent 24 h period of sEV production in a final volume of 400 mL in SpF and in 200 mL in STBr. **(A)** Representative images of the 1.4E7 spheroids obtained after 72 h in either SpF or STBr. Scale bar = 100 µm. Evolution of the spheroids **(B)** concentration and **(C)** size. **(D)** Number of cells per spheroid throughout the culture duration. **(E)** Schematic representation of the flat paddle impeller in the SpF and the dual 30° three-segment pitched-blade impellers in the STBr, along with their corresponding flow regime patterns. **(F)** Violin plot showing the size of the 1.4E7 spheroids after 72 h of culture in SpF and STBr at 8.5 W/m^3^, including the corresponding Kolmogorov length scale. **(G)** Growth curves (continuous line) and viability assessed using trypan blue exclusion (dashed line). **(H)** Cell density at 48 h prior to medium renewal. **(I)** Viability at 72 h assessed based on the quantification of dead cells, extrapolated from LDH measurements in cell culture supernatants. **(J)** Percentage of free cells in cultures. **(K)** Interfacial area per mL, calculated from spheroid and free cell size and concentration. Results are expressed as means ± SD. Numbers of biological replicates from independent experiments were as follows: SpF (n = 4) and STBr (n = 8). Statistical analyzes were performed using the Mann-Whitney test (*p < 0.05; **p < 0.01; ****p < 0.0001).

Representative images of the 1.4E7 spheroids obtained in the 0.5 L SpF and the STBr at the end of the sEV production phase are shown in Figure 5A. Throughout the process, significantly greater numbers of spheroids were obtained in the STBr at any time point (Figure 5B), with these spheroids being smaller in size (Figure 5C). At the end of the process, in STBr, 3572 spheroids ±943, of 92 µm ± 7 were obtained per mL, with each spheroid containing 84 cells ±17 (Figure 5D), compared to 327 spheroids ±90 (p < 0.01) of 230 µm ± 23 (p < 0.01) per mL, each containing 1137 cells ±361 (p < 0.01) in SpF. As illustrated in Figure 5E, a tangential flow regime was achieved in the SpF equipped with a flat paddle impeller, while the dual 30° three-segment pitched-blade impellers of the STBr generated an axial flow regime. These striking differences in the vessels and, notably, the impellers geometry, affected the flow regime, thereby impacting spheroid formation.

The hydrodynamic characteristics of both systems are summarized in Table 1. No major difference was observed concerning the Re (6173 in 0.5 L SpF *vs.* 4795 in STBr) and similar *Θm* were determined (7.4 s in the SpF *vs*. 6 s in the STBr). As *P/V* remained constant, both systems achieved comparable mean shear stress. The calculated shear rate $\langle \dot{\gamma }\rangle$ was approximately 110 s^−1^ while the $\langle \lambda_{K}\rangle$ was equal to ∼80 µm in both systems. Since all spheroids in the SpF were larger than the $\langle \lambda_{K}\rangle$ , they likely experienced shear stress (Cherry, 1993), whereas in the STBr, ∼25% of spheroids remained unaffected by shear stress (Figure 5F). However, these global measures neglect the heterogeneity of energy dissipation. Specifically, the energy dissipation rate and associated shear stress, are higher and maximal near the impellers. As the *υ*_*tip*_ was nearly 2-fold higher in the STBr (0.41 m/s *vs.* 0.23 m/s), and since there were two impellers, shear stress was likely locally higher in STBr compared to SpF.

Regarding cell performance and viability, slight cell proliferation was observed in the 0.5 L SpF despite the absence of FBS, whereas no proliferation was noted in the STBr (Figure 5G, H). We observed similar and high cell viability (>95%) using trypan blue exclusion in both stirred systems throughout the process (Figure 5G). A slightly higher LDH release was detected in the STBr, resulting in a calculated cell viability of 93.6% ± 1.5 in the SpF and 90.1% ± 3.1 in the STBr at 72 h (p < 0.05) (Figure 5I). A slightly higher percentage of free cells was found in the STBr at 48 h: 19.8% ± 4.6 in the STBr *vs.* 13.8% ± 3.5 in the SpF (p < 0.05) and this difference remained after medium renewal (13.4% ± 3.9 in the STBr *vs.* 5.0% ± 1.4 in the SpF, p < 0.05). However, at the end of the sEV production phase, the percentage of free cells was not significantly different anymore (Figure 5J). Lastly, we estimated that the more numerous, smaller spheroids in the STBr increased the cell exchange surface, referred as interfacial area. The spheroids generated in the STBr represented 92 mm^2^/mL ± 16 compared to 54 mm^2^/mL ± 12 in the SpF at 72 h, while no significant difference was observed in the interfacial area of the free cells (Figure 5K). Regarding cell metabolism, no major difference was observed between both systems (Supplementary Figure 1).

### 3.4 Characterization of 1.4E7-derived sEV

**FIGURE 6:**
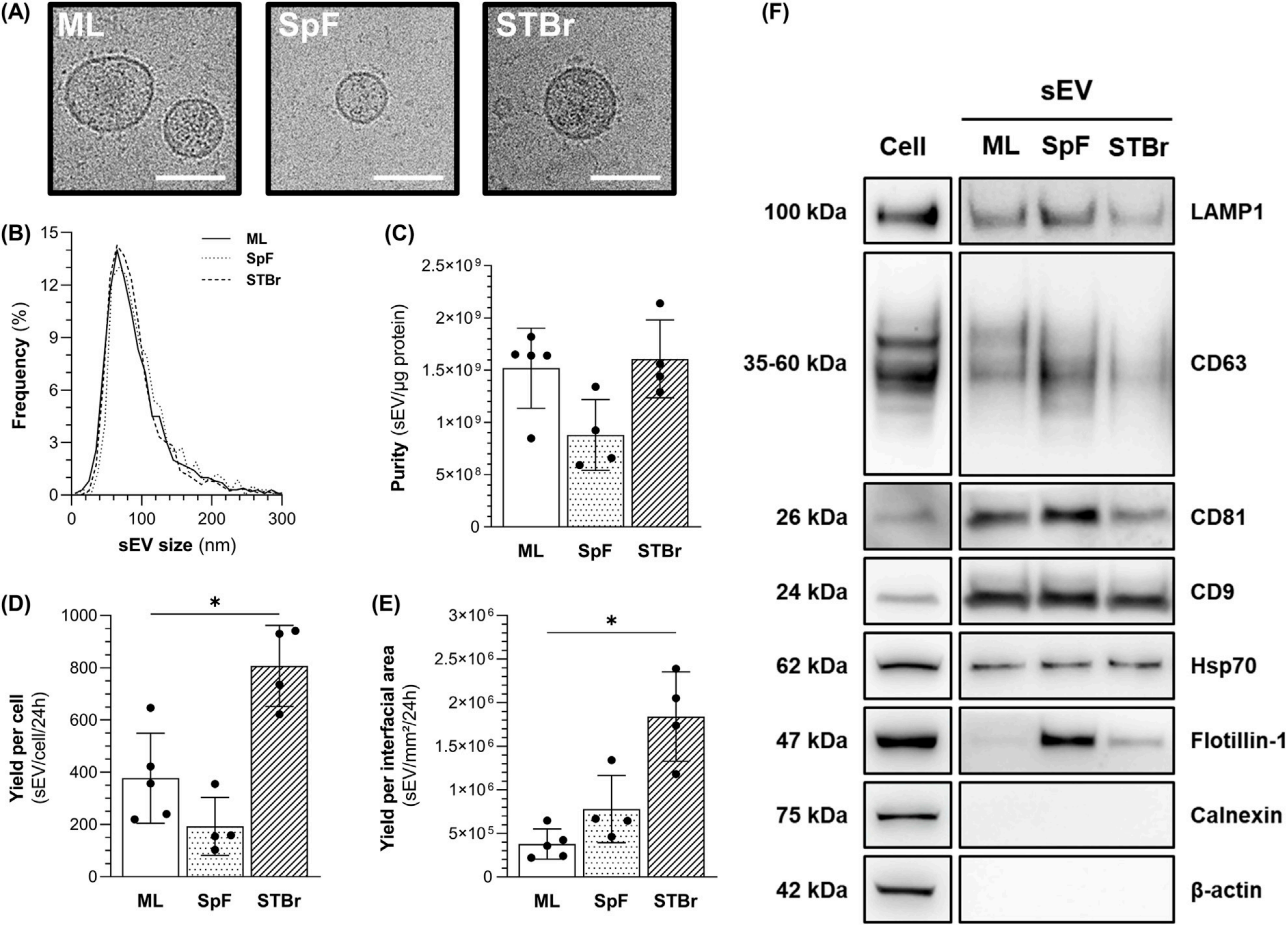
Characterization of 1.4E7-derived sEV from monolayers (ML), 0.5 L spinner flasks (SpF), or stirred-tank bioreactors (STBr) cultures. **(A)** Representative cryo-transmission electron microscopy images of 1.4E7-derived sEV from two independent experiments/group. Scale bar = 200 nm. **(B)** Size distribution of tetraspanin-labeled particles measured using nanoparticle tracking analysis. **(C)** Purity of sEV batches expressed as the ratio of tetraspanin-positive particles per µg of protein. 1.4E7-derived sEV yields at 24 h expressed as the number of tetraspanin-positive particles per **(D)** producing cell or **(E)** interfacial area. Results are expressed as means ± SD. Numbers of biological replicates from independent experiments were as follows: ML (n = 5), SpF (n = 4) and STBr (n = 4). Statistical analyzes were performed using the Mann-Whitney test (*p < 0.05). **(F)** Representative western blot of sEV-associated proteins, ML (n = 4), SpF (n = 3), STBr (n = 3).

1.4E7-derived sEV were isolated from the conditioned medium of cells grown as ML (T-flask) or spheroids in SpF and STBr for 24 h in the absence of FBS, as described. Data regarding sEV production volumes, cell concentrations and sEV yields per condition are summarized in the Supplementary Table 1. Cryo-transmission electron microscopy revealed round-shaped membrane particles in all conditions (Figure 6A, see Supplementary Figure 2 for uncropped images). NTA analysis of tetraspanin-labeled particles also showed a homogenous sEV size distribution across conditions (Figure 6B). The downstream process, combining TFF and SEC, allowed for the recovery of highly pure sEV samples of up to 2 × 10^9^ sEV/µg proteins (Figure 6C). The highest sEV yield was obtained from STBr cultures, which was 2-fold higher than that from ML cultures, while the lowest yield was observed in SpF cultures (Figure 6D). We hypothesize that spheroid size and concentration may significantly contribute to these differences in sEV yields. Interestingly, when normalized to the interfacial area, the lowest sEV yield was observed in ML cultures, while production in stirred systems increased the yield (2-fold in SpF and 5-fold in STBr) (Figure 6E). To confirm the sEV identity of the isolated particles, and in accordance with the MISEV2023 recommendations (Welsh et al., 2024), western blots were performed to detect sEV-enriched proteins and to ensure the absence of proteins associated with irrelevant intracellular compartments (e.g*.*, calnexin). As shown in Figure 6F, the sEV isolated from each culture mode displayed typical expression of membrane-bound proteins (Lamp1, CD63, CD81, and CD9) and cytosolic proteins (Hsp70 and flotillin-1). Notably, the expression of flotillin-1 was higher in stirred systems, particularly in SpF, as observed in four independent western blots. Finally, sEV from each condition did not express β-actin (cytoskeleton) or calnexin (endoplasmic reticulum), confirming the purity of the isolated sEV.

**FIGURE 7:**
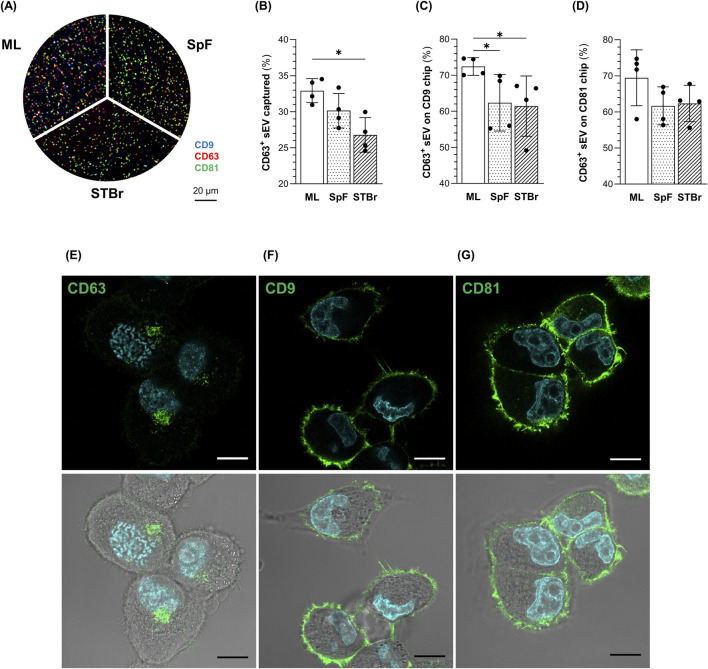
Quantification of CD63 ^+^ 1.4E7-derived sEV from monolayers (ML), 0.5 L spinner flasks (SpF), or stirred-tank bioreactors (STBr) cultures and localization of tetraspanins CD63, CD9 and CD81 in 1.4E7 cells. **(A)** Representative images of sEV captured on CD63 ExoView™ human tetraspanin chips and labeled with fluorescent antibodies directed against CD9 (blue), CD63 (red), and CD81 (green). **(B)** Percentage of sEV captured on CD63 chips relative to the total number of sEV captured on CD9, CD63, and CD81 chips. **(C)** Percentage of CD63^+^ sEV (including CD63^+^, CD63+/CD81+, CD63+/CD9+, and CD63+/CD9+/CD81+ labeled) captured on CD9 chips or **(D)** CD81 chips. Results are expressed as means ± SD and are based on 4 samples per group from two independent experiments. Statistical analyzes were performed using the Mann-Whitney test (*p < 0.05). Confocal imaging of the cellular localization of **(E)** CD63, **(F)** CD9 and **(G)** CD81 in 1.4E7 cells, with (bottom) of without (top) white light. Nuclei were counterstained with Hoechst 33342. Scale bar = 10 µm.

In addition, an ExoView™ array was performed to quantify the amount of 1.4E7-derived sEV expressing the CD9, CD63, and CD81 tetraspanins from each sEV production method. Representative images of sEV captured on CD63 chips are shown in Figure 7A. CD81 was the most highly expressed tetraspanin in each sEV sample (Supplementary Figure 3A). Interestingly, the percentage of sEV captured on CD63 chips was significantly higher in ML cultures than in the STBr (Figure 7B). In addition, the percentage of CD63^+^ sEV captured on CD9 chips was higher in ML cultures compared to both stirred systems (Figure 7C). A similar profile was observed with CD81 chips, although the differences were not significant (Figure 7D). In contrast to CD63, culture mode did not affect the expression of CD9 and CD81 (Supplementary Figures 3A–F).

Immunostaining of CD63, CD9, and CD81 was performed on 1.4E7 cells grown as ML to determine their cellular localization. CD63 was predominantly expressed at intracellular locations (Figure 7E), whereas CD9 (Figure 7F) and CD81 (Figure 7G) were almost exclusively found on the plasma membrane. These observations suggest that sEV expressing CD63 primarily originate from subcellular compartments as exosomes, while sEV lacking CD63 but expressing CD9 or CD81 derive from the plasma membrane as ectosomes.

Altogether, these data indicate that highly pure and intact sEV expressing typical EV markers were obtained from each culture system. Interestingly, a lower proportion of CD63^+^ sEV was isolated from spheroid cultures in stirred systems, suggesting an enrichment of plasma membrane-derived sEV.

## 4 Discussion

The production of clinical-grade EV faces significant challenges, including the development of standardized and scalable upstream and downstream processes, and precise characterization of both the producing cells and the EV, in compliance with regulatory guidelines. In this study, we aimed to investigate the scalability of a serum-free EV production process from anchorage-dependent cells cultured as spheroids. Using the 1.4E7 human β cell line, we compared the attributes of spheroid-derived sEV produced in stirred systems to those produced in a ML reference process. Additionally, we explored the impact of hydrodynamics on cell and spheroid fate and investigated scale-up strategies to standardize spheroid generation.

Regardless of FBS supplementation, 3D culture promoted the self-aggregation of 1.4E7 cells into compact, well-defined, and highly viable spheroids, without substrate limitations or too high accumulation of toxic metabolic wastes. Culturing 1.4E7 cells as spheroids induced a shift towards oxidative pathway respiration, indicating a more efficient use of glucose for energy production (Vander Heiden et al., 2009). 1.4E7 cells exhibited significant growth rate reduction when cultured as spheroids, regardless of FBS supplementation. Therefore, the process we developed included an initial expansion phase using ML cultures with FBS, while sEV production was carried out in STBr using spheroid cultures in a serum-free, chemically defined medium. The largest spheroids were obtained after 72 h of culture in SpF at a *P/V* of 2.6 W/m^3^, with an average diameter of 248 µm ± 20. Gas, nutrient, and metabolic waste diffusion with the extracellular environment appeared to be sufficient at these sizes (Debruyne et al., 2024).

Stirred systems have been proposed as effective methods for producing large quantities of size-adjusted spheroids compared to static culture approaches (Liu et al., 2021). Additionally, STBr are the preferred culture systems for closely monitoring cell culture parameters and scaling up processes. They are extensively used for producing biological medicines, including recombinant proteins, vaccines, and viral vectors. However, these processes typically rely on the culture of cells in suspension, and little is known about the scalability of spheroid cultures and the impact of stirred systems on the attributes of spheroid-derived EV. We demonstrated that spheroid formation in stirred systems was dependent on the *P/V*, and maintaining this parameter constant was an adapted scale-up parameter to standardize spheroid size and morphology, in accordance with previous studies (Manstein et al., 2021; Petry and Salzig, 2022). We then transferred the process from SpF to a fully controlled STBr. As expected, significant differences were observed in STBr cultures compared to SpF, primarily due to the marked differences in vessel geometry especially the impeller design. Olmer et al. previously reported that at a constant agitation rate, the size of induced pluripotent stem cell (iPSC) spheroids depended on the angles of the pitched-blade impeller segments used in their STBr setup. However, the specific N_P_ of each impeller and the consequent *P/V* of each culture were not reported (Olmer et al., 2012). At a constant *P/V*, Petry and Salzig demonstrated significant differences in the formation of INS-1 spheroids in shaking flasks and in the INFORS HT Labfors 5^®^ STBr, depending on whether the system was equipped with a single 30° or 45° three-segment pitched-blade impeller, or a single six-segment Rushton impeller (Petry and Salzig, 2022).

1.4E7 spheroids generated in the STBr appeared slightly less compact, with a less-defined morphology and a slightly higher percentage of free cells compared to the ones produced in SpF. This suggests reduced spheroid formation efficiency in the STBr setup, potentially due to the dual-pitched blade impellers. Other studies used single impellers-equipped STBr, and reported the formation of more homogenous and well-defined spheroids (Manstein et al., 2021; Petry and Salzig, 2022; Olmer et al., 2012). Overall, these findings suggest that dual impellers may not be ideal for spheroid cultures, primarily since they double the regions where maximum shear stress is exerted.

Future work will focus on investigating the impact of different impeller designs on spheroid formation, morphology, cell viability, and metabolism. The process we developed in STBr maintained high cell viability and a metabolic profile similar to SpF on short period of time. By improving the cell-interfacial area with the medium, this process enhanced the sEV yield. However, several challenges remain to be addressed in order to increase sEV yield per batch, particularly by extending the production period to allow for multiple sEV harvests.

Highly pure and intact sEV expressing typical EV markers were obtained from each production method. Compared to the ML reference process, spheroid culture in SpF resulted in a 2-fold decrease in sEV yield per cell, while culture in STBr led to a 2-fold increase. We hypothesize that spheroid size plays a crucial role in sEV release, as previously suggested (Kim et al., 2018; Rovere et al., 2023). Specifically, the fewer but larger spheroids generated in SpF may have retained high amount of sEV, while the more numerous and smaller spheroids from STBr provided a greater interfacial area with the culture medium. Notably, when expressing sEV yields per unit of interfacial area, we observed a 2-fold increase in SpF and a 5-fold increase in STBr compared to ML cultures.

In accordance with immunofluorescence analyzes performed here, and previous studies identifying CD63 as a genuine exosome marker (Kowal et al., 2016; Mathieu et al., 2021), the lower frequency of CD63^+^ sEV suggests an increased release of plasma membrane-derived EV (ectosomes) rather than endosomal EV (exosomes) under stirred conditions. Although a better characterization of sEV phenotype would require additional analyzes, our results suggest that production under stirred conditions could be of particular interest for cytosolic and plasma membrane-bound compounds loading into sEV.

Based on Kolmogorov’s theory of turbulence (Kolmogorov, 1941), spheroids generated in both stirred systems might have experienced shear stress as they were larger than the calculated $\langle \lambda_{K}\rangle$ ≈ 80 µm. Although turbulence was not fully achieved in the 0.125 L SpF and STBr, it is assumed that Kolmogorov’s theory of isotropic turbulence applied (Nienow et al., 2016). Shear stress has been widely described as a factor that enhances EV production (reviewed by Thompson and Papoutsakis, 2023). Additionally, since the outer layer of the spheroids was more exposed to shear stress than the core, we hypothesize that a significant amount of sEV was released as ectosomes due to surface erosion from the outer layer (Petry and Salzig, 2022; Petry and Salzig, 2021), which might explain the lower CD63 expression observed in stirred system-derived sEV.

Applying moderate shear stress is a technique used to produce large quantities of functional MSC-derived EV (Eichholz et al., 2020; Berger et al., 2021; Guo et al., 2021; Kang et al., 2022; Barekzai et al., 2023). However, extensive or prolonged shear stress may affect cell performance and viability (Croughan et al., 1987; Cherry, 1993; Espina et al., 2023), as well as stem cell differentiation (Vining and Mooney, 2017), and its impact on the final qualitative properties of sEV must be considered. Notably, protecting spheroids from shear stress appears to be very challenging in conventional STBr. Indeed, since $\langle \lambda_{K}\rangle$ is proportional to the *P/V*, it can be reduced by applying a lower *P/V*. However, because spheroid size depends on the *P/V*, bigger spheroids would be produced under low *P/V* conditions, increasing the shear stress they undergo. Conversely, smaller spheroids could be generated at higher *P/V* but the resulting shear stress would also be increased. In our studies in SpF, after 3 days of culture at 2.6, 8.6 or 20.4 W/m^3^, spheroids of 287, 248, and 176 µm mean size were obtained, respectively. The corresponding $\langle \lambda_{K}\rangle$ were 108, 80, and 64 µm, respectively. Thus, in each condition, spheroids might undergo shear stress as their diameter is three times the calculated $\langle \lambda_{K}\rangle$ .

Cytoprotective agents might offer a promising solution to preserve spheroid cultures from mechanical and shear stress; however, conventional surfactant cytoprotectants such as Pluronic-F68 may not be suitable for EV production, as they incorporate into biological membranes (Gigout et al., 2008) and might affect EV attributes. Other additives could be investigated to protect spheroids from hydrodynamic constraints while preserving EV release and isolation, including albumin, methylcellulose, or polyethylene glycol (Wu, 1995). Another strategy explored is the encapsulation of spheroids in hydrogel to avoid excessive agglomeration, protect the cells from shear stress, and provide support to avoid anoïkis, while allowing the free diffusion of gases, nutrients, and metabolic wastes. Using microfluidic devices, Fattahi et al*.* encapsulated human iPSCs in polyethylene glycol, enabling the formation and culture of spheroids in SpF (Fattahi et al., 2021). More recently, Cohen et al*.* encapsulated human iPSCs in alginate microcapsules internally coated with Matrigel. Human iPSCs successfully self-organized into cysts and were cultured up to 10 L in STBr for 7 days, while maintaining their pluripotency and achieving higher cell viability and proliferation than ML, microcarriers, or spheroid cultures (Cohen et al., 2023). Furthermore, sEV have already been successfully isolated from HeLa spheroids grown in peptide hydrogel under static conditions (Thippabhotla et al., 2019). Additionally, Han et al*.* cultured MSC in gelatin methacryloyl hydrogel under static conditions and demonstrated a higher neuroprotective effect of the produced sEV compared to a conventional ML process (Han et al., 2022). However, depending on the hydrogel used and its cross-linking, the free diffusion of EV might be impaired.

Finally, the production of spheroid-derived sEV might be achieved in systems with specific designs to mitigate hydrodynamic constraints, such as the recently proposed vertical wheel bioreactor (VWR). Unlike traditional impeller systems, the VWR bioreactor employs a wheel mechanism, which is believed to generate both radial and axial flows to achieve a uniform hydrodynamic force distribution and create a low shear-stress environment (Dang et al., 2021). High yields of functional sEV have been obtained in the VWR from MSC grown on microcarriers (de Almeida Fuzeta et al., 2020; Jalilian et al., 2022; Jeske et al., 2023; Otahal et al., 2024) as well as from iPSC spheroids (Muok et al., 2024) and forebrain spheroids (Liu et al., 2024). However, a scale-up to industrial-scale production (several m^3^) is not yet feasible as the VWR’s maximum volume available is currently 80 L.

In this study, we used the 1.4E7 cell line, described as a β cell line expressing insulin (McCluskey et al., 2011). Unfortunately, we detected neither insulin mRNA nor the protein in cells cultured under low and high glucose conditions. Also, apart from *PDX1*, we did not detect either mRNAs from other major β cells antigens such as *GAD65* (glutamate decarboxylase 65), *ZnT8* (proton-coupled Zinc antiporter SLC30A8), *IAPP* (islet amyloid polypeptide), *PTPRN2* (Receptor-type tyrosine-protein phosphatase N2) and *CMGA* (chromogranin A) in cells cultured as ML or spheroids in serum-containing medium. The absence of insulin and other major β cell antigens was further confirmed by two independent proteomic analyzes performed on low-passage cells from two separate vials obtained from the supplier. Since the 1.4E7 cell line did not exhibit a β cell phenotype, the therapeutic potential of 1.4E7-derived EV for type 1 diabetes is compromised.

Nevertheless, the bioprocess solution outlined here could be applied to human β cell lines expressing key β cell markers and glucose responsiveness, such as the EndoC-βH1-3 cell line, for which large-scale culture still needs to be established (Scharfmann et al., 2019). Another promising approach is the differentiation of stem cells into β cells (Hogrebe et al., 2023). Lastly, our study highlights the interest in producing batches of EV in conditions compatible with upscaling to ensure comparable properties of EV. It also opens the way towards cultivating in larger STBr, the 1.4E7 cell line as a model of spheroids, to develop downstream processes for EV purification from larger volumes of culture. This is of major interest since for sEV, as for any biopharmaceutical production, each process parameter has to be carefully considered and addressed to ensure the final product’s quality and quantity attributes, bearing in mind that “the process is the product.”

## Data Availability

The original contributions presented in the study are included in the article/Supplementary Material, further inquiries can be directed to the corresponding author.

## Supplementary material

SUPPLEMENTARY FIGURE 1 Metabolite profile of the 1.4E7 cells grown in production medium in 0.5 L spinner flasks (SpF) or stirred-tank bioreactors (STBr) at a *P/V* of ∼8.5 W/m^3^. Cells were initially cultured for 48 h to form spheroids, after which ∼85% of the production medium was renewed for a subsequent 24 h period of sEV production. **(A)** Glucose, **(B)** lactate, **(C)** glutamine and **(D)** ammonium concentrations measured in the cell culture supernatants. **(E)** Glucose and **(G)** glutamine specific consumption rates (*q*_*s*_), **(F)** lactate and **(H)** ammonium specific production rates (*q*_*p*_). **(I)** Lactate-to-glucose and **(J)** ammonium-to-glutamine metabolic yields (*Y*_*Lac/Glc*_ and *Y*_*NH4/Gln*_, respectively). **(K)** Cell-to-glucose and **(L)** cell-to-glutamine growth yields (*Y*_*cells/Glc*_ and *Y*_*cells/Gln*_, respectively). Results are expressed as means ± SD. Numbers of biological replicates from independent experiments were as follows: SpF (n = 4) and STBr (n = 8). Statistical analyzes were performed using the Mann-Whitney test (**p < 0.01).

SUPPLEMENTARY FIGURE 2 Uncropped raw images of cryo-microscopy. Scale bar = 200 nm.

SUPPLEMENTARY FIGURE 3 ExoView™ analyzes of 1.4E7-derived sEV from monolayers (ML), 0.5 L spinner flasks (SpF), or stirred-tank bioreactors (STBr) cultures. **(A)** Percentage of sEV captured on CD81 chips relative to the total number of sEV captured on CD9, CD63, and CD81 chips. **(B)** Percentage of CD81^+^ sEV (including CD81^+^, CD81+/CD63+, CD81+/CD9+, and CD63+/CD9+/CD81+ labeled) captured on CD63 chips or **(C)** CD9 chips. **(D)** Percentage of sEV captured on CD9 chips relative to the total number of sEV captured on CD9, CD63, and CD81 chips. **(E)** Percentage of CD9^+^ sEV (including CD9^+^, CD9+/CD63+, CD9+/CD81+, and CD63+/CD9+/CD81+ labeled) captured on CD63 chips or **(F)** CD81 chips. Results are expressed as mean ± SD and are based on 4 samples per group from two independent experiments.

SUPPLEMENTARY TABLE 1 Description of the sEV batches produced in the different systems. sEV were purified from cell culture medium after 24 h as described. Tetraspanin-labeled particles were analyzed using the Zetaview as described, allowing enumeration of total sEV. Means ± SD are reported. Unconditioned medium (371 mL) was purified as control of the process. In this sample, the particle yield was 3.1 × 107/mL, but no signal was obtained following tetraspanin labeling, or staining with the CMDR™ (a lipidic fluorescent dye that binds all cell membranes, independently of the species).

## Abbreviations

DO, dissolved oxygen; FBS, fetal bovine serum; HFB, hollow-fiber bioreactor; LDH, lactate dehydrogenase; $\langle \lambda_{K}\rangle$ , mean Kolmogorov length scale; ML, monolayer; NTA, nanoparticle tracking analysis; PDT, population doubling time; P/V, volumetric power input; qP, specific production rate; qS, specific consumption rate; Re, Reynolds number; SEC, size exclusion chromatography; sEV, small extracellular vesicles; SpF, spinner flask; STBr, stirred-tank bioreactor; TFF, tangential flow filtration; µmax, maximal growth rate; υtip, impeller tip speed; VWR, vertical wheel bioreactor; YLac/Glc, Lactate-to-Glucose metabolic yield; YNH4/Gln, ammonium-to-glutamine metabolic yield.
