# Supplementary material for: Scalability of spheroid-derived small extracellular vesicles production in stirred systems
Source: Front Bioeng Biotechnol. 2025 Apr 29;13:1516482. doi: 10.3389/fbioe.2025.1516482 (PMC12069995; doi:10.3389/fbioe.2025.1516482)
Supplement: Supplementary file 3 [file Table1.docx]

**Supplementary Table 1**: Description of the sEV batches produced in the different systems.

Description of the sEV batches produced in the different systems. sEV were purified from cell culture medium after 24 h, as described. Tetraspanin-labeled particles were analyzed using the Zetaview as described, allowing enumeration of total sEV. Means ± SD are reported. Unconditioned medium (371 mL) was purified as control of the process. In this sample, the particle yield was 3.1×10^7^/ml, but no signal was obtained following tetraspanin labeling, or staining with the CMDR™ (a lipidic fluorescent dye that binds all cell membranes, independently of the species).

| **Parameters** | **ML** | **SpF** | **STBr** |
| --- | --- | --- | --- |
| Number of batches | 5 | 4 | 4 |
| Production volume (mL) | 422 ± 66 | 392 ± 36 | 357 ± 25 |
| Alive cell concentration (×10^6^ cells/mL) | 0.73 ± 0.13 | 0.38 ± 0.046 | 0.32 ± 0.011 |
| sEV mode size (nm) | 72.5 ± 5.5 | 68.2 ± 5.0 | 72.9 ± 1.9 |
| sEV yield  per volume (×10^7^ /ml/24h) | 28 ± 15 | 7.5 ± 4.7 | 26 ± 5.3 |
| per cell (/cell/24h) | 337 ± 172 | 193 ± 111 | 807 ± 156 |
| per interfacial area (×10^5^ /mm²/24h) | 3.8 ± 1.7 | 7.8 ± 3.9 | 18.0 ± 5.1 |
| sEV purity (×10^8^ /µg protein) | 15.0 ± 3.8 | 8.8 ± 3.4 | 16.0 ± 3.7 |
| Total amount of sEV per batch (×10^10^) | 13.0 ± 8.0 | 3.0 ± 2.2 | 9.2 ± 2.2 |
| Total amount of protein per batch (µg) | 80.3 ± 39.0 | 36.4 ± 23.0 | 58.4 ± 13.5 |
| Protein yield  per cell (×10^-9^ µg /cell/24h) | 250 ± 76 | 170 ± 170 | 510 ± 92 |
| per volume (µg/ml/24h) | 0.18 ± 0.07 | 0.09 ± 0.05 | 0.15 ± 0.05 |
