# Supplementary figures and images for: Scalability of spheroid-derived small extracellular vesicles production in stirred systems
Source: Front Bioeng Biotechnol. 2025 Apr 29;13:1516482. doi: 10.3389/fbioe.2025.1516482 (PMC12069995; doi:10.3389/fbioe.2025.1516482)

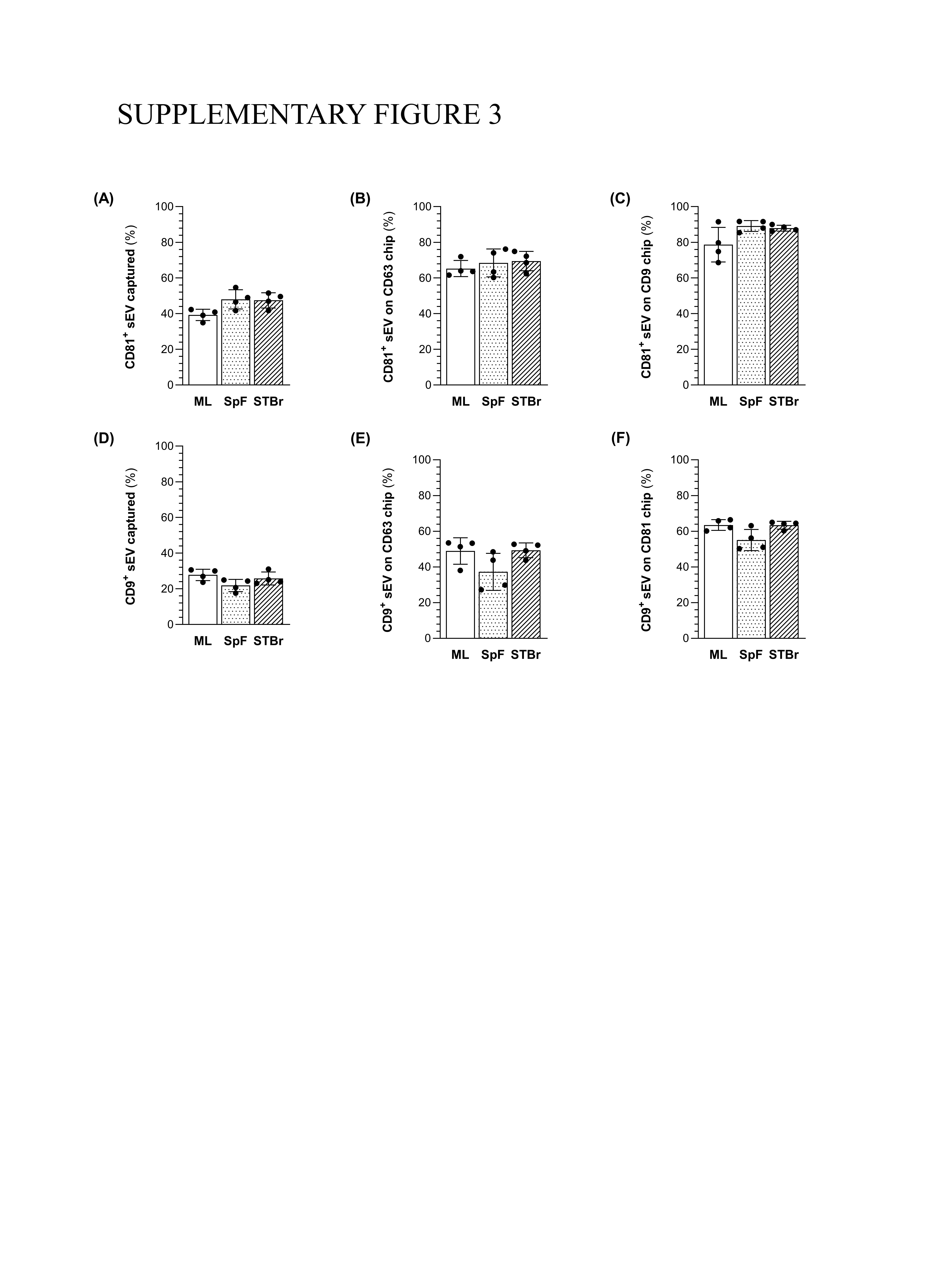

Supplement: Supplementary file 4 [file Image3.tif]

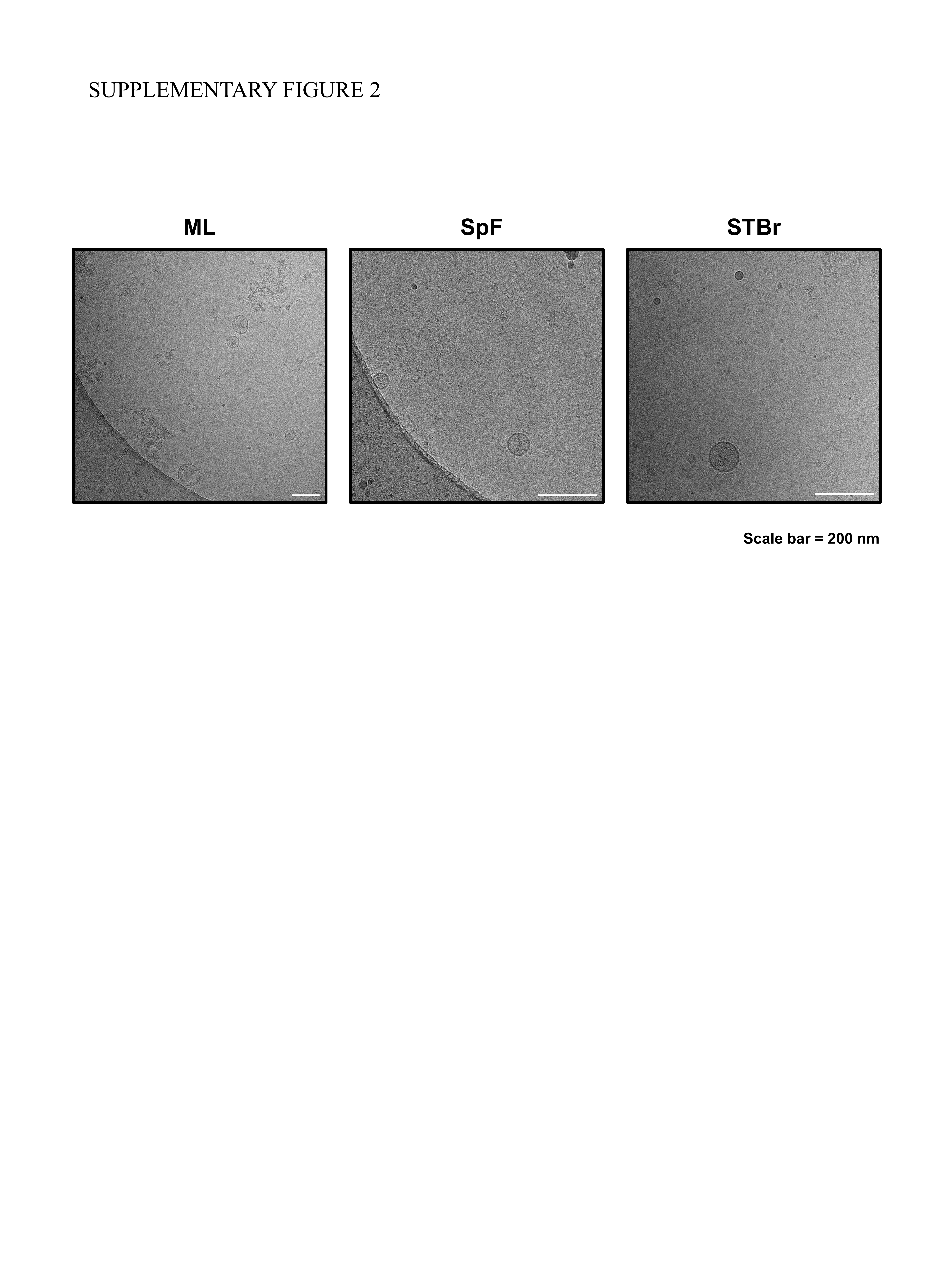

Supplement: Supplementary file 5 [file Image2.tif]

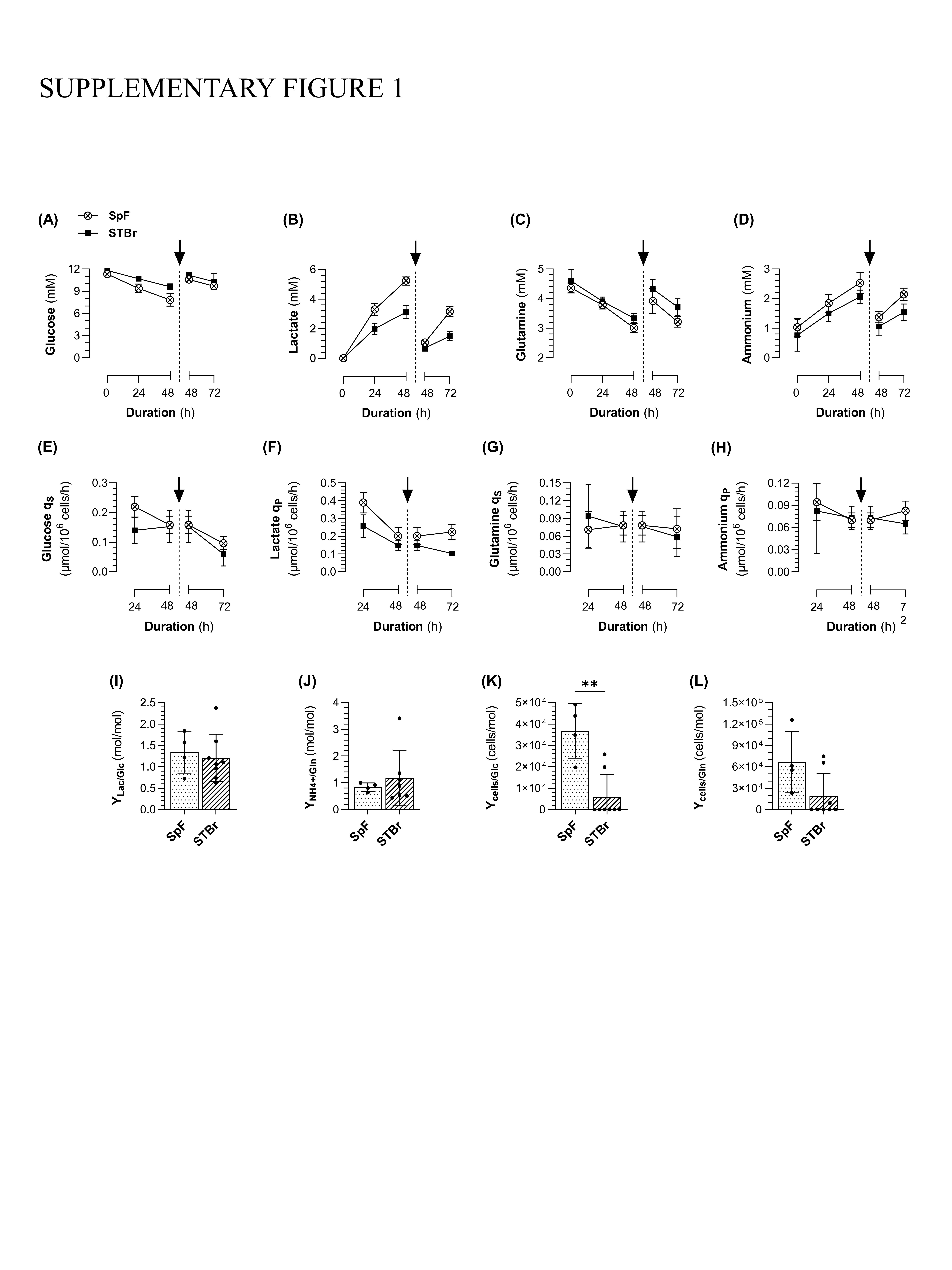

Supplement: Supplementary file 6 [file Image1.tif]
